# Associations between SNPs and immune-related circulating proteins in schizophrenia

**DOI:** 10.1038/s41598-017-12986-0

**Published:** 2017-10-03

**Authors:** Man K. Chan, Jason D. Cooper, Stefanie Heilmann-Heimbach, Josef Frank, Stephanie H. Witt, Markus M. Nöthen, Johann Steiner, Marcella Rietschel, Sabine Bahn

**Affiliations:** 1 Department of Chemical Engineering and Biotechnology, University of Cambridge, Cambridge, United Kingdom; 2 Institute of Human Genetics, University of Bonn School of Medicine & University Hospital Bonn, Sigmund-Freud-Strasse 25, D-53127 Bonn, Germany; 3 Department of Genomics, Life & Brain Center, University of Bonn, Sigmund-Freud-Strasse 25, D-53127 Bonn, Germany; 4 Department of Genetic Epidemiology in Psychiatry, Central Institute of Mental Health, Faculty of Medicine Mannheim, University of Heidelberg, J5, 65189 Mannheim, Germany; 5 Department of Psychiatry, University of Magdeburg, Magdeburg, Germany

## Abstract

Genome-wide association studies (GWAS) and proteomic studies have provided convincing evidence implicating alterations in immune/inflammatory processes in schizophrenia. However, despite the convergence of evidence, direct links between the genetic and proteomic findings are still lacking for schizophrenia. We investigated associations between single nucleotide polymorphisms (SNPs) from the custom-made PsychArray and the expression levels of 190 multiplex immunoassay profiled serum proteins in 149 schizophrenia patients and 198 matched controls. We identified associations between 81 SNPs and 29 proteins, primarily involved in immune/inflammation responses. Significant SNPxDiagnosis interactions were identified for eight serum proteins including Factor-VII[rs555212], Alpha-1-Antitrypsin[rs11846959], Interferon-Gamma Induced Protein 10[rs4256246] and von-Willebrand-Factor[rs12829220] in the control group; Chromogranin-A[rs9658644], Cystatin-C[rs2424577] and Vitamin K-Dependent Protein S[rs6123] in the schizophrenia group; Interleukin-6 receptor[rs7553796] in both the control and schizophrenia groups. These results suggested that the effect of these SNPs on expression of the respective proteins varies with diagnosis. The combination of patient-specific genetic information with blood biomarker data opens a novel approach to investigate disease mechanisms in schizophrenia and other psychiatric disorders. Our findings not only suggest that blood protein expression is influenced by polymorphisms in the corresponding gene, but also that the effect of certain SNPs on expression of proteins can vary with diagnosis.

## Introduction

Schizophrenia is a heritable and heterogeneous disorder likely to be affected by environmental factors. The combined evidence from genetic, epidemiological, transcriptomic and proteomic studies has now converged at alterations in metabolic, neurotrophic and prominently, immune/inflammatory processes in schizophrenia. The largest genome-wide association study (GWAS) conducted has identified 108 schizophrenia associated genetic loci involved in glutamatergic neurotransmission and synaptic plasticity and, importantly in immune processes^1^. Epidemiological and transcriptomic studies have long hinted at a role for immune dysregulation in schizophrenia^2–5^. Blood-based protein biomarker studies have also demonstrated changes in immune/inflammatory processes in prodromal and drug-naïve patients, from which candidate diagnostic and prognostic biomarker panels have been reported^6^. In addition, clinical trials have shown initial encouraging therapeutic effects associated with add-on anti-inflammatory medication in schizophrenia patients^7^.

Despite the convergence of evidence, direct links between the genetic and proteomic findings have as yet not been established for schizophrenia. While genetics may provide insights into the biological mechanisms underpinning disease susceptibility, proteomics can provide functional molecular evidence linked to disease manifestation. Although GWAS studies have ‘implicated’ a number of candidate genes, these studies have shown that most of the associations are to genomic regions (‘loci’). For most of these loci, it is not certain which exact gene is affected^8^. In addition, because almost all the schizophrenia-associated single nucleotide polymorphisms (SNPs) have been found to be located within non-coding regions of genes, elucidation and interpretation of the biological basis for the genetic associations remains challenging^8^.

With this in mind, we attempted for the first time to investigate associations between 190 serum proteins implicated in several psychiatric disorders^9–12^ including schizophrenia^6,13–16^ and SNPs located within genes encoding for the measured proteins in 149 schizophrenia patients and 198 matched controls. The SNPs will be analysed by the custom-made PsychArray, which was developed by Illumina in collaboration with the Psychiatric Genomic Consortium (PGC). It assesses approximately 270,000 tag SNPs, over 250,000 rare and low-frequency exonic variants and approximately 50,000 custom markers selected based on evidence from prior genetic studies of psychiatric illnesses including schizophrenia, major depressive, bipolar and autism-spectrum disorders^17^.

## Materials and Methods

### Clinical samples

A total of 347 individuals were recruited consecutively from the department of Psychiatry, University of Magdeburg, Germany including 198 controls and 149 schizophrenia patients (109 first-onset antipsychotic drug-naïve and 40 antipsychotic drug treated). Diagnosis of schizophrenia was performed by psychiatrists using the Diagnostic and Statistical Manual of Mental Disorders (DSM-IV)^18^. Information on antipsychotic medication use was confirmed by direct contact with the treating family physicians and relatives along with consultations regarding detailed histories of psychotropic medication use prior to hospitalization. Controls were matched with the patient group for age, gender and body mass index (BMI) (Table 1) and were recruited from a database of blood donors at the Institute of Transfusion Medicine at the University of Magdeburg and, some were students and staff at the University. Exclusion criteria included chronic illnesses such as diabetes, cardiovascular disease, immune and autoimmune disorders, infections, treatment with immune- suppressive or -modulating drugs or antibiotics, other neuropsychiatric or neurological disorders (multiple sclerosis, epilepsy, mental retardation), chronic (terminal) diseases affecting the brain (cancer, hepatic and renal insufficiency), alcohol or drug addiction, organic psychosis/organic affective syndromes, severe trauma, other psychiatric and non-psychiatric co-morbidity. Medication was administered after completion of diagnostic evaluation as appropriate. Informed written consent was given by all participants and the study protocols, analysis of samples and test methods were approved by the local Institutional Ethics Review Board and were in compliance with the Standards for Reporting of Diagnostic Accuracy^19^.

**Table 1:** Demographic characteristics.

	**Controls (198)**	**Schizophrenia**			**P-value**
		**All (149)**	**First onset (109)**	**Chronic (40)**	
**Age** ^W^	36|35 (11) [18–65]	37|36 (11) [16–66]	36|34 (11) [16–66]	39|41 (9) [22–53]	0.5025
**BMI (kg/m** ^**2**^ **)** ^W^	26|25 (4) [18–41]	26|26 (5) [17–41]	25|24 (5) [17–37]	29|28 (5) [21–41]	0.3940
**Gender** ^F^					
Female	94 (47%)	64 (43%)	40 (37%)	22 (55%)	0.3286
Male	104 (53%)	85 (57%)	69 (63%)	18 (45%)	
**Previous antipsychotic medication**					
Yes	NA	40 (27%)	0 (0%)	40 (100%)	NA
No	NA	109 (73%)	109 (100%)	0 (0%)	

Numerical values are shown as Mean|Median (Standard Deviation) [Min-Max].

Key: **F**, Fisher’s exact test; **W**, Wilcoxon test; **P-values** are calculated using the control group as the reference (i.e. difference between the patient and control group).

### Serum sample preparation

Serum sample collection and preparation followed strict standard operating protocols, as described previously^13^. Briefly, blood samples were collected from all subjects between 8:00 and 12:00 hours in the morning into S-Monovette 7.5 mL serum tubes (Sarstedt; Numbrecht, Germany). The samples were left to clot at room temperature for 2 hours and then centrifuged at 4000 × g for 5 minutes. The resulting supernatants were stored at −80 °C in Low Binding Eppendorf tubes (Hamburg, Germany).

### Multiplex immunoassay analysis and Quality Control

The Multi-Analyte Profiling immunoassay platform (DiscoveryMAP) was used to measure the concentrations of 190 proteins in patient sera. The proteins measured were mainly involved in immune/inflammatory, endocrine and metabolic processes previously implicated in several neurological/psychiatric disorders^9–12^ including schizophrenia^6,13–16^, depression^20,21^ and bipolar disorder^22^. All assays were conducted in the Clinical Laboratory Improved Amendments (CLIA)–certified laboratory at Myriad-RBM (Austin, TX, USA; described previously^13^). All serum samples were stored at −80 °C until analysis. Data were quality control (QC) assessed and pre-processed using R (http://www.R-project.org/)^23^, as described previously^6^. Briefly, proteins with greater than 30% missing values were excluded and values below or above the detection limits were imputed by the minimum and maximum detected values, respectively. Data were log_2_-transformed to stabilise variance. Sample outliers were examined using principal components analysis (PCA)^24^ and through inspection of quantile-quantile (Q-Q) plots.

### Genotyping and Quality Control

Blood DNA samples from all participants were genotyped using the Illumina Infinium PsychArray v1.0 (Illuminia Inc, San Diego, California, USA) at the Department of Genomics at the Life and Brain Centre, University of Bonn. Data was quality control (QC) assessed using PLINK v1.07^25^ and R (http://www.R-project.org/)^23^, as described previously^26^. Per-individual QC involved exclusion of samples with **(1)** over 3% missing genotypes (no samples); **(2)** abnormal heterozygosity rate ±3 standard deviations from the mean (7 samples); **(3)** related or duplicated samples (7 samples) identified through identity by -state and -descend sharing analysis on an linkage disequilibrium-pruned set of SNPs (for each pair related or duplicated individuals, the individual with lower genotyping completeness was excluded); and, **(4)** individuals of non-European ancestry (2 samples) identified by combining study genotypes with genotypes from HapMap3 data with the following population codes: CEU (Utah residents with Northern and Western European ancestry), YRI (Yoruba in Ibadan, Nigeria), CHB (Han Chinese in Beijing, China), JPT (Japanese in Tokyo, Japan). Per-SNP QC involved exclusion of SNPs **(1)** with over 5% missing genotype (2348); **(2)** showing excessive deviation from Hardy-Weinberg equilibrium (*P* < 10^−3^) in the control group (800); **(3)** significantly different missing genotype rates between cases and controls (*P* < 10^−5^) (0); and, **(4)** with a very low minor allele frequency (MAF) of less than 1% (278912).

### SNP selection

The genotype data were subjected to gene annotation using the biomaRt package^27^ in R to identify the SNPs located within genes encoding for the measured proteins. These SNPs were selected for downstream linear regression analysis.

### Statistical analysis

All statistical analyses were performed in R (http://www.R-project.org/)^23^. Linear regression analyses were carried out to identify association between SNPs and the corresponding proteins. For each regression model, each SNP was individually included as the predictor variable (continuous variable coded 0, 1, 2 counting the number of major alleles) along with covariates, diagnosis (binary case/control status) and a SNP x Diagnosis interaction term to identify case/control specific SNP and protein associations. Protein concentration was modelled as the continuous outcome variable. The covariates were age, gender, BMI and antipsychotic medication. Regression diagnostics were examined to ensure that all the model assumptions were met including check for residual normality, data linearity, independence and homoscedasticity and, exclusion of high leverage points, outliers and influential values. False discovery rate was controlled according to Benjamini and Hochberg^28^. To account for regression model stability and robustness of findings, bootstrap resampling was repeated 1000 times for each test^29^. Given the exploratory nature of the study, SNP-protein associations were accepted as significant if adjusted *P*-values < 0.05 or if *P*-values < 0.05 in over 70% of 1000 bootstrap samples. SNPxDiagnosis results were strictly only accepted as significant following multiple correction at adjusted P-value < 0.05.

## Results

The demographic characteristics of the study cohort are summarised in Table 1. The patient and control groups were matched for age, gender and BMI. The mean age and BMI of patients was 36 and 26, respectively and for controls 37 and 26, respectively. The percentage of males/females was 57%/43% for patients and 53%/47% for controls.

### SNP and protein expression association

**Figure 1:**
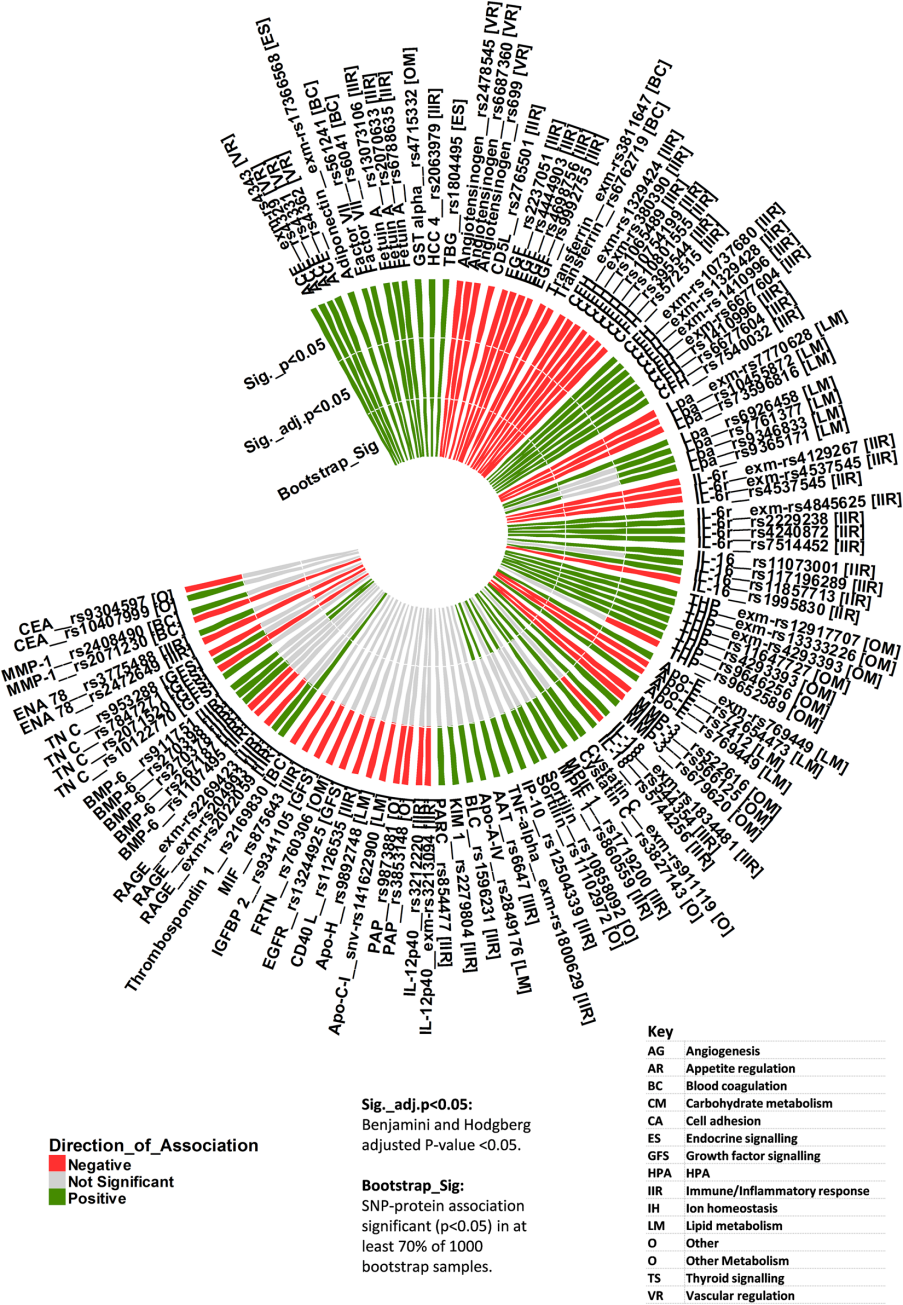
Polar histogram showing the significant SNP-protein associations. Key: A positive direction of association (β) indicates that a higher major allele copy number is associated with a higher protein level in blood. A negative direction of association (−β) indicates that a higher major allele copy number is associated with a lower protein level in blood.

In total, 149 of the 190 proteins measured by the multiplex immunoassay platform survived QC. Following genetic data QC, 308,263 of the original 588,454 SNPs were left for analysis and sample size was reduced to 331 (189 controls and 142 schizophrenia patients). Of these, we found that 632 SNPs were located within 128 genes that encode for 132 of the measured proteins (for minor allele frequencies of SNPs, see Supplementary Table 1). This represented 89% SNP coverage for the 149 proteins surviving QC. Linear regression analysis showed that 115 SNPs were associated with 45 proteins (P-value < 0.05) (Table 2 and Fig. 1). Of these, associations between 81 SNPs and 29 proteins survived multiple testing (adjusted P-value < 0.05) and/or bootstrap resampling. These 29 proteins were involved in several biological functions including ***immune/inflammatory response (14)*** [Complement Factor H, Interleukin-6 receptor (IL-6r), Epithelial-Derived Neutrophil-Activating Protein 78 (ENA-78), Fetuin-A, Interleukin-16 (IL-16), Epidermal Growth Factor (EGF), CD5L, Receptor for advanced glycosylation end products (RAGE), Interleukin-18 (IL-18), Chemokine CC-4 (HCC-4), Bone Morphogenetic Protein 6 (BMP-6), Tumor Necrosis Factor alpha (TNF-alpha), Interferon gamma Induced Protein 10 (IP-10), Myeloid Progenitor Inhibitory Factor 1 (MPIF-1)], ***blood coagulation (3)*** [Factor VII, Serotransferrin, Matrix Metalloproteinase-1 (MMP-1)], ***lipid metabolism (2)*** [Apolipoprotein E (Apo-E), Apolipoprotein(a) (Lpa)], ***other metabolic processes (3)*** [Tamm-Horsfall Urinary Glycoprotein (THP), Matrix Metalloproteinase-3 (MMP-3), Glutathione S-Transferase alpha (GST-alpha)], ***endocrine or growth factor signalling (3)*** [Adiponectin, Thyroxine-Binding Globulin (TBG), Tenascin-C (TN-C)], ***vascular regulation (2)*** [Angiotensin-Converting Enzyme (ACE), Angiotensinogen] and, ***other (2)*** [Cystatin-C, Sortilin]. All directions of association between the SNPs and their corresponding proteins were consistent, except for associations with six proteins including CFH, Lpa, IL-6r, IL-16, Apo-E and MMP-3. This finding suggests that these SNPs may have differential regulatory effects on protein expression.

**Table 2:** Association of 115 SNPs with expression of 45 proteins.

Protein	Protein Abbrev	Gene	SNP	Chr	β	P-value	Adjusted P-value	Bootstrap Sig. (%)
Adiponectin	Adiponectin	ADIPOQ	exm-rs17366568	3	**0.32**	1.99E-03	1.98E-02	87
Alpha-1-Antitrypsin	AAT	SERPINA1	rs6647	14	**0.10**	2.95E-02	1.79E-01	51
Angiotensin-Converting Enzyme	ACE	ACE	exm-rs4343	17	**0.41**	4.44E-19	3.53E-17	100
			rs4329	17	**0.40**	4.93E-18	2.61E-16	100
			rs4331	17	**0.40**	2.56E-18	1.48E-16	100
			rs4362	17	**0.40**	5.90E-19	4.17E-17	100
Angiotensinogen	Angiotensinogen	AGT	rs2478545	1	*−1.76*	3.19E-14	1.01E-12	100
			rs6687360	1	*−1.37*	6.39E-10	1.23E-08	100
			rs699	1	*−1.25*	3.76E-09	6.65E-08	100
Apolipoprotein A-IV	Apo-A-IV	APOA4	rs2849176	11	**0.16**	2.55E-02	1.65E-01	59
Apolipoprotein C-I	Apo-C-I	APOC1	snv-rs141622900	19	*−0.19*	2.16E-02	1.44E-01	58
Apolipoprotein E	Apo-E	APOE	exm-rs769449	19	**0.30**	1.10E-03	1.18E-02	81
			rs769449	19	**0.30**	1.10E-03	1.18E-02	81
			rs72654473	19	−*0.34*	1.10E-03	1.18E-02	89
			rs7412	19	−*0.49*	5.66E-06	8.18E-05	99
Apolipoprotein H	Apo-H	APOH	rs9892748	17	−*0.10*	2.87E-02	1.79E-01	57
Apolipoprotein(a)	Lpa	LPA	exm-rs7770628	6	−*0.76*	1.46E-04	1.82E-03	95
			rs73596816	6	−*2.13*	4.88E-06	7.22E-05	100
			rs10455872	6	−*3.02*	4.43E-13	1.34E-11	100
			rs6926458	6	**0.91**	3.19E-05	4.32E-04	98
			rs7761377	6	**0.55**	6.25E-03	5.30E-02	72
			rs9346833	6	**0.40**	4.28E-02	2.31E-01	57
			rs9365171	6	**0.55**	6.97E-03	5.84E-02	71
B Lymphocyte Chemoattractant	BLC	CXCL13	rs1596231	4	**0.39**	4.40E-02	2.35E-01	54
Bone Morphogenetic Protein 6	BMP-6	BMP6	rs1107495	6	−0.33	3.32E-02	1.98E-01	63
			rs267187	6	−0.26	3.35E-02	1.98E-01	64
			rs270378	6	**0.28**	2.92E-02	1.79E-01	62
			rs270398	6	**0.39**	7.53E-03	5.99E-02	82
			rs911751	6	**0.31**	7.24E-03	5.90E-02	84
Carcinoembryonic Antigen	CEA	CEACAM5	rs10407999	19	**0.32**	2.59E-02	1.67E-01	62
			rs9304597	19	−*0.34*	2.00E-02	1.39E-01	66
CD40 Ligand	CD40 L	CD4LG	rs1126535	23	−*0.25*	3.64E-02	2.08E-01	53
CD5L	CD5L	CD5L	rs2765501	1	−*0.16*	1.53E-04	1.87E-03	94
Chemokine CC-4	HCC 4	CCL16	rs2063979	17	**0.24**	2.92E-03	2.81E-02	75
Complement Factor H	CFH	CFH	exm-rs380390	1	−*0.36*	1.60E-12	4.44E-11	100
			rs395544	1	−*0.36*	1.50E-12	4.33E-11	100
			exm-rs1329424	1	−*0.35*	1.91E-11	4.34E-10	100
			rs10754199	1	−*0.35*	1.91E-11	4.34E-10	100
			rs10801555	1	−*0.35*	1.91E-11	4.34E-10	100
			rs572515	1	−*0.35*	1.91E-11	4.34E-10	100
			rs1065489	1	−*0.24*	4.86E-03	4.23E-02	86
			exm-rs1329428	1	**0.49**	2.26E-23	2.99E-21	100
			rs7540032	1	**0.49**	2.26E-23	2.99E-21	100
			exm-rs10737680	1	**0.49**	3.29E-23	2.99E-21	100
			exm-rs1410996	1	**0.49**	3.16E-23	2.99E-21	100
			rs1410996	1	**0.49**	3.16E-23	2.99E-21	100
			exm-rs6677604	1	**0.76**	4.65E-66	1.48E-63	100
			rs6677604	1	**0.76**	4.65E-66	1.48E-63	100
Cystatin-C	Cystatin C	CST3	exm-rs911119	20	**0.08**	7.94E-03	6.23E-02	73
			rs3827143	20	**0.09**	4.46E-03	3.94E-02	80
Epidermal Growth Factor	EGF	EGF	rs2237051	4	−*0.18*	8.40E-04	9.54E-03	95
			rs4444903	4	−*0.21*	6.00E-05	7.94E-04	99
			rs4698756	4	−*0.22*	1.20E-04	1.53E-03	99
			rs9992755	4	−*0.20*	4.03E-04	4.84E-03	97
Epidermal Growth Factor Receptor	EGFR	EGFR	rs13244925	7	−*0.06*	2.18E-02	1.44E-01	67
Epithelial-Derived Neutrophil-Activating Protein 78	ENA 78	CXCL5	rs3775488	4	**0.17**	1.76E-02	1.24E-01	61
			rs2472649	4	−*0.64*	3.57E-12	9.46E-11	100
Factor VII	Factor VII	F7	rs561241	13	**0.37**	1.18E-05	1.66E-04	97
			rs6041	13	**0.37**	2.12E-05	2.94E-04	99
Ferritin	FRTN	FTH1	rs760306	11	−*0.34*	2.80E-02	1.76E-01	56
Fetuin-A	Fetuin A	AHSG	rs13073106	3	**0.21**	2.84E-10	5.65E-09	100
			rs2070633	3	**0.21**	1.45E-10	3.17E-09	100
			rs6788635	3	**0.22**	2.03E-10	4.29E-09	100
Glutathione S-Transferase alpha	GST alpha	GSTA1	rs4715332	6	**0.47**	3.49E-03	3.17E-02	84
Insulin-like Growth Factor-Binding Protein 2	IGFBP 2	IGFBP2	rs9341105	2	−*0.16*	3.74E-02	2.11E-01	52
Interferon gamma Induced Protein 10	IP-10	CXCL1	rs12504339	4	**0.19**	1.32E-02	9.45E-02	76
Interleukin-12 Subunit p40	IL-12p40	IL12B	exm-rs3213094	5	−*0.20*	4.22E-02	2.29E-01	48
			rs3212220	5	−*0.20*	4.22E-02	2.29E-01	48
Interleukin-16	IL-16	IL16	rs117196289	15	**0.30**	4.88E-02	2.54E-01	48
			rs11073001	15	**0.16**	5.76E-03	4.95E-02	73
			rs11857713	15	**0.51**	2.32E-10	4.76E-09	100
			rs1995830	15	−*0.17*	3.38E-03	3.16E-02	81
Interleukin-18	IL-18	IL18	exm-rs1834481	11	**0.17**	1.30E-03	1.36E-02	91
			rs544354	11	−*0.15*	4.62E-02	2.45E-01	53
			rs5744256	11	**0.17**	1.30E-03	1.36E-02	91
Interleukin-6 receptor	IL-6r	IL6R	exm-rs4129267	1	−*0.44*	1.36E-18	8.68E-17	100
			exm-rs4537545	1	−*0.42*	1.41E-17	6.41E-16	100
			rs4537545	1	−*0.42*	1.06E-17	5.21E-16	100
			rs4240872	1	**0.30**	1.46E-07	2.27E-06	100
			exm-rs4845625	1	**0.31**	1.07E-09	1.99E-08	100
			rs2229238	1	**0.32**	9.63E-08	1.53E-06	100
			rs7514452	1	**0.32**	6.69E-08	1.09E-06	100
Kidney Injury Molecule-1	KIM 1	HAVCR1	rs2279804	5	**0.17**	4.22E-02	2.29E-01	52
Macrophage Migration Inhibitory Factor	MIF	MIF	rs875643	22	−*0.22*	1.92E-02	1.34E-01	58
Matrix Metalloproteinase-1	MMP-1	MMP1	rs2071230	11	−*0.44*	2.54E-03	2.49E-02	88
			rs2408490	11	**0.27**	3.66E-02	2.08E-01	52
Matrix Metalloproteinase-3	MMP-3	MMP3	rs522616	11	−*0.21*	3.03E-03	2.88E-02	88
			rs566125	11	−*0.31*	5.39E-04	6.35E-03	90
			rs679620	11	**0.33**	2.70E-08	4.51E-07	100
Myeloid Progenitor Inhibitory Factor 1	MPIF 1	CCL23	rs1719200	17	**0.23**	1.11E-02	8.14E-02	65
			rs860559	17	**0.15**	1.05E-02	7.93E-02	71
Prostatic Acid Phosphatase	PAP	ACPP	rs3853148	3	−*0.12*	2.68E-02	1.70E-01	61
			rs9873881	3	−*0.14*	2.53E-02	1.65E-01	58
Pulmonary and Activation-Regulated Chemokine	PARC	CCL18	rs854477	17	**0.14**	2.08E-02	1.40E-01	61
Receptor for advanced glycosylation end products	RAGE	AGER	exm-rs2022059	6	**0.76**	6.12E-04	7.08E-03	87
			exm-rs204993	6	**0.19**	3.23E-02	1.94E-01	60
			exm-rs2269423	6	−*0.15*	4.86E-02	2.54E-01	50
Serotransferrin	Transferrin	TF	exm-rs3811647	3	−*0.10*	1.40E-03	1.44E-02	91
			rs6762719	3	−*0.09*	3.45E-03	3.17E-02	85
Sortilin	Sortilin	SORT1	rs10858092	1	**0.13**	8.59E-03	6.66E-02	73
			rs11102972	1	**0.12**	2.06E-02	1.40E-01	65
Tamm-Horsfall Urinary Glycoprotein	THP	UMOD	exm-rs12917707	16	**0.62**	1.59E-15	6.31E-14	100
			exm-rs13333226	16	**0.58**	1.59E-14	5.33E-13	100
			exm-rs4293393	16	**0.60**	3.40E-15	1.20E-13	100
			rs11647727	16	**0.54**	1.24E-15	5.28E-14	100
			rs4293393	16	**0.60**	2.19E-15	8.18E-14	100
			rs9646256	16	**0.37**	2.81E-09	5.10E-08	100
			rs9652589	16	−*0.14*	2.94E-02	1.79E-01	57
Tenascin-C	TN C	TNC	rs10122770	9	**0.34**	7.45E-03	5.99E-02	68
			rs2071520	9	−*0.17*	3.62E-03	3.24E-02	83
			rs7847271	9	**0.21**	3.61E-02	2.08E-01	53
			rs953288	9	−*0.14*	1.08E-02	8.06E-02	75
Thrombospondin-1	Thrombospondin 1	THBS1	rs2169830	15	−*0.17*	4.05E-02	2.26E-01	48
Thyroxine-Binding Globulin	TBG	SERPINA7	rs1804495	23	**0.18**	1.99E-03	1.98E-02	84
Tumor Necrosis Factor alpha	TNF-alpha	TNF	exm-rs1800629	6	**0.30**	9.70E-03	7.44E-02	79

Chr, chromosome; β, regression coefficient estimates; Italic, negative association; Bold, positive association; Underline, Adjusted P-values < 0.05 or SNP-protein association significant (P-value < 0.05) in at least 70% of 1000 bootstrap samples.

### SNP x Diagnosis interaction effect on protein expression

**Figure 2:**
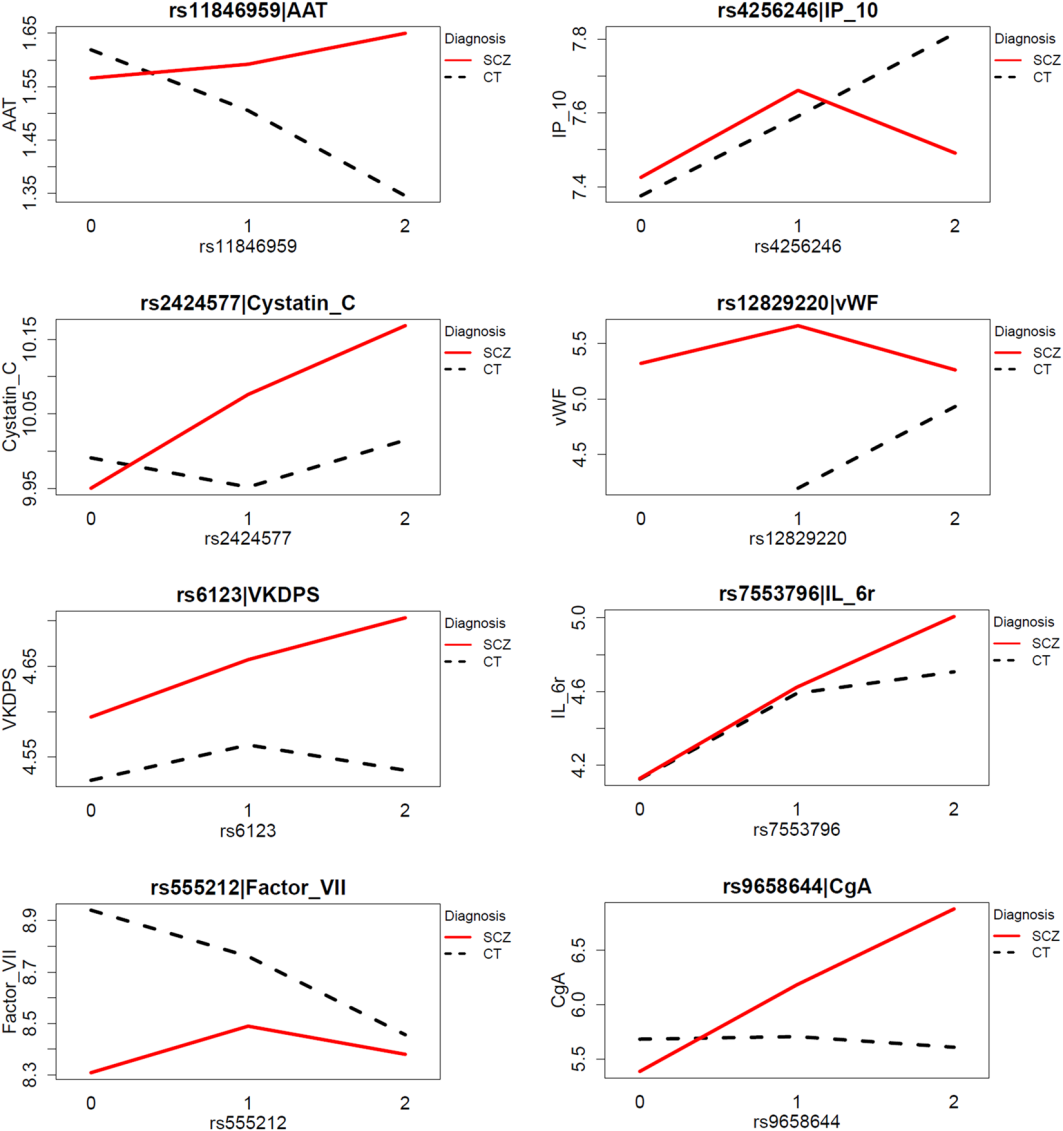
Interaction plots showing significant SNP and protein expression associations where SNP x Diagnosis interaction was significant.

A total of 21 SNPs showed significant SNP x Diagnosis interactions for 19 proteins suggesting that the effect of these SNPs on expression of the respective proteins varies with diagnosis (Table 3). Separate analysis stratified by diagnosis showed that seven SNPs were associated with seven proteins in the control group, another seven SNPs showed associations with seven proteins in the schizophrenia group and two SNPs were associated with two proteins in both the control and schizophrenia groups (for the complete list of significant SNP and protein associations stratified by diagnosis, see Supplementary Table 2). Approximately half of these SNP-protein associations survived multiple testing including rs555212 (Factor VII) (β = −0.3, adj.P = 3.07E-05]), rs11846959 (Alpha-1-Antitrypsin; AAT) (β = −0.19, adj.P = 0.004), rs4256246 (IP-10) (β = 0.23, adj.P = 0.038) and rs12829220 (von Willebrand Factor; vWF) (β = 0.75, adj.P = 0.006) in the control group; rs9658644 (Chromogranin-A; CgA) (β = 0.77, adj.P = 0.008), rs2424577 (Cystatin-C) (β = 0.09, adj.P = 0.009) and rs6123 (Vitamin K-Dependent Protein S; VKDPS) (β = 0.09, adj.P = 0.034) in the schizophrenia group; rs7553796 (IL-6r) in both the control (β = 0.28, adj.P = 9.44E-07) and schizophrenia (β = 0.43, adj.P = 3.33E-10) groups (Table 3, Fig. 2, Supplementary Figure 1). Figure 2 shows that while an increasing number of major alleles of rs7553796 is associated with increasing levels of the IL-6r protein in blood in both groups, schizophrenia patients with two copies of the major allele (homozygous for the major allele) have significantly higher levels of the IL-6r protein compared to controls homozygous for the major allele. A positive β indicates that a higher number of major alleles is associated with higher protein expression in blood. A negative β indicates that a higher number of major alleles is associated with a lower protein level in blood.

**Table 3:** Significant SNP x Diagnosis interaction and results stratified by diagnosis.

Protein	Protein Abbrev	Gene	SNP	Chr	Interaction P-value	Controls			Schizophrenia		
						β	P-value	Adjusted P-value	β	P-value	Adjusted P-value
Interleukin-6 receptor	IL-6r	IL6R	rs7553796	1	3.88E-02	**0.28**	5.64E-08	9.44E-07	**0.43**	5.23E-12	3.33E-10
Chromogranin-A	CgA	CHGA	rs2295396	14	2.40E-03	**0.53**	0.016	0.105	−0.54	0.027	0.210
Factor VII	Factor VII	F7	rs555212	13	1.52E-02	−*0.30*	2.03E-06	3.07E-05	−0.04	0.521	0.831
Alpha-1-Antitrypsin	AAT	SERPINA1	rs11846959	14	3.72E-04	−*0.19*	3.29E-04	0.004	0.08	0.052	0.322
Interferon gamma Induced Protein 10	IP-10	CXCL1	rs4256246	4	9.85E-03	**0.23**	0.004	0.038	−0.21	0.096	0.441
von Willebrand Factor	vWF	VWF	rs12829220	12	1.01E-02	**0.75**	0.006	0.047	−0.23	0.383	0.745
Tenascin-C	TN C	TNC	rs7043308	9	1.69E-02	**0.19**	0.011	0.079	−0.09	0.312	0.689
E-Selectin	E Selectin	SELE	rs3917419	1	7.99E-03	−*0.11*	0.032	0.177	0.13	0.137	0.533
Leptin	Leptin	LEP	rs28954099	7	5.27E-03	−*0.60*	0.041	0.205	0.75	0.080	0.398
Chromogranin-A	CgA	CHGA	rs9658644	14	2.45E-03	−0.13	0.529	0.790	**0.77**	0.001	0.008
Cystatin-C	Cystatin C	CST3	rs2424577	20	3.32E-02	0.02	0.463	0.750	**0.09**	0.001	0.009
Vitamin K-Dependent Protein S	VKDPS	PROS1	rs6123	3	2.30E-02	−0.01	0.809	0.932	**0.09**	0.003	0.034
Chromogranin-A	CgA	CHGA	rs750678	14	1.39E-02	−0.13	0.489	0.756	**0.57**	0.011	0.104
Interleukin-12 Subunit p40	IL-12p40	IL12B	rs2569254	5	1.23E-02	0.12	0.265	0.569	−*0.29*	0.014	0.127
B Lymphocyte Chemoattractant	BLC	CXCL13	rs142545798	4	2.86E-02	−0.32	0.563	0.815	**1.60**	0.016	0.135
CD5L	CD5L	CD5L	rs16839299	1	4.73E-02	0.07	0.435	0.742	−*0.35*	0.039	0.264
Matrix Metalloproteinase-9	MMP-9	MMP9	rs13925	20	1.35E-02	−0.08	0.363	0.684	0.18	0.053	0.328
Resistin	Resistin	RETN	rs80035917	19	2.92E-02	−0.12	0.193	0.494	0.11	0.261	0.656
Epidermal Growth Factor Receptor	EGFR	EGFR	rs2072454	7	3.53E-02	−0.03	0.183	0.487	0.03	0.348	0.718
Stem Cell Factor	SCF	KITLG	exm-rs995030	12	4.49E-02	−0.12	0.072	0.294	0.07	0.371	0.737
Prolactin	PRL	PRL	rs78697234	6	3.26E-02	−0.66	0.118	0.387	0.20	0.727	0.911

Table showing the significant SNP and protein expression associations stratified by diagnosis for the regression models where SNP x Diagnosis interaction was significant. A significant SNP x Diagnosis interaction indicates that effect of SNPs on protein expression varies with diagnosis (binary case/control status).

Key**:** Interaction P-value, SNP x Diagnosis interaction significance.

## Discussion

We investigated the association between SNPs genotyped using the PsychArray and the expression levels of 190 serum proteins in 149 schizophrenia patients and 198 matched controls. The hypothesis was that protein expression levels for a given individual are associated with SNPs in the corresponding gene.

We found that 632 SNPs were located within 128 genes that encode for 132 of the measured serum proteins. Linear regression analysis identified associations between 81 SNPs and 29 proteins that survived corrections for multiple testing and/or bootstrap resampling. Interestingly, more than half of these proteins could be associated with immune and inflammation responses. The remaining proteins were found to be related to a number of pathways ranging from blood coagulation, metabolism, endocrine signalling to vascular regulation.

As a next step, we investigated the SNP x diagnosis interaction. When the effect of a SNP on protein-level differs between patients and controls (e.g., present in one group and absent in the other), this indicates a difference in the biological regulation of the protein level in patients compared to controls. Furthermore, insights can be obtained into whether the involvement of a protein-biomarker in disease development involves disease-specific pathways (Supplementary Figure 2). We found eight serum proteins with a significant interaction which survived multiple testing following analysis stratified by diagnosis, namely rs555212 (Factor VII), rs11846959 (AAT), rs4256246 (IP-10) and rs12829220 (vWF) in the control group; rs9658644 (CgA), rs2424577 (CST3) and rs6123 (VKDPS) in the schizophrenia group; rs7553796 (IL-6r) in both the control and schizophrenia groups. Four out of these eight proteins (AAT, IP10, vWF and IL6r) were involved in immune function/inflammatory processes. This observation aligns with the finding that schizophrenia associations are enriched at enhancers that are active in tissues linked to immune function^1^. Importantly, all of the implicated proteins have previously been repeatedly reported to be differentially expressed in serum or plasma of schizophrenia patients^6,13–16^. Some of these proteins such as Factor VII, vWF, CgA, AAT and IL-6r have also been found to be altered or predict development of schizophrenia in ultra-high risk or pre-onset individuals^6,30^. However, to our knowledge only SNPs associated with IL-6r, CgA, vWF and AAT have previously been reported in schizophrenia. The strength of our study was that through genotyping using the PsychArray and profiling of circulating protein using the DiscoveryMAP platform, we have attempted to demonstrate a functional link between expression of protein biomarkers previously implicated in schizophrenia and schizophrenia related SNPs located within genes encoding for the measured proteins.

The IL-6r gene has been investigated extensively in previous genetic studies. However, results have not been consistent. While a previous genetic association study of IL-6r reported a significant association of rs2228145 C allele (Ala allele) with schizophrenia^31^, others failed to find significant differences in allele or genotype distribution between patients and controls^32^. Another study investigated promoter polymorphism of another IL-6r rs4845617 but found no significant association with schizophrenia in Taiwan^33^. Kapelski and colleagues recently reported a significant association of rs2228145 and rs4537545 with schizophrenia^34^. Rafiq and colleagues found that the minor allele T of rs4537545 accounted for approximately 20% of the variation in circulating IL-6r levels and individuals homozygous for the minor allele of the rs4537545 had a doubling of IL-6r levels compared to the major allele homozygous group^35^. Our results are in line with this finding. We found that individuals homozygous for the minor allele of rs4537545 and its exome SNP exm-rs4537545 had the highest IL-6r levels compared to individuals homozygous for the major allele, which had the lowest IL-6r levels (Table 2). However, rs4537545 x diagnosis interaction was not significant suggesting that the effect of this SNP on IL-6r expression does not vary with diagnosis (patient or control). Analysis stratified by diagnosis also showed significant association between the SNPs (rs4537545 and exm-rs4537545) and IL-6r expression in the control and patient groups separately demonstrating further that these SNPs are associated with IL-6r expression regardless of diagnosis (Supplementary Table 2). In addition to this finding, we also demonstrated that another SNP, rs7553796 located within the IL-6r gene was associated with increasing levels of the IL-6r protein in blood in both the control and schizophrenia groups (Table 3). A significant rs7553796 x diagnosis interaction and subsequent analysis stratified by diagnosis showed that while increasing number of the major allele is associated with increasing IL-6r levels in both groups, schizophrenia patients homozygous for the major allele had significantly higher levels of the IL-6r protein compared to controls homozygous for the major allele (Fig. 2). This finding suggests the possibility of differential regulation of protein expression in schizophrenia patients based on the major allele copy number of rs7553796.

The other interesting protein that has been studied extensively is CgA. This protein is widely expressed in secretory granules throughout the central nervous system and in endocrine tissue and is co-released with several neurotransmitters^36^. CgA has calcium binding and neuromodulatory properties and is a potent microglial activator resulting in neurotoxicity mediated through the secretion of glutamate, TNF alpha and nitric oxide which in turn induces mitochondrial stress and apoptosis^36^. Changes in the expression of CgA protein in schizophrenia have been reproducibly shown in post-mortem^37^ frontal cortex and pituitary, CSF^38,39^ and serum^6,37,40^. Biochemical studies have also demonstrated a reduction of CgA immunoreactivity in the prefrontal cortex of schizophrenic patients^41^. Alterations in CSF CgA levels have also been shown in a range of neurodegenerative disorders, Parkinson’s^42^ and Alzheimer’s^43^ disease. Allelic-association studies in Chinese patients have associated single-nucleotide polymorphisms at the chromogranin B (CHGB) locus with schizophrenia^44^. Genetic linkage studies in Japanese schizophrenia patients have implicated a genomic region near the CHGB locus on chromosome 20^45^. Subsequent Japanese studies have reported significant associations between schizophrenia and the CHGB gene, which belongs to the same family as CHGA^46^. More recently, studies showing associations between rs9658635 at promoter region and the haplotype of rs9658635–rs729940 in the *CHGA* gene with schizophrenia have also emerged^36^. Through SNP x diagnosis interaction analysis, we found that the effect of the rs9658644 on expression of the CgA protein varied with diagnosis (Table 3). Analysis stratified by diagnosis showed that an increase in number of major alleles of rs9658644 was significantly associated with an increase in expression of the CgA protein in the schizophrenia patient group. In the control group, the major allele was not associated with CgA protein levels in blood (Fig. 2). These results suggest differential regulation of CgA protein expression in patients based on number of rs9658644 major alleles.

The SERPINA1 gene, which encodes for the AAT protein has also been implicated in schizophrenia. Association studies demonstrated that the rs1303 displayed different genotype pattern distributions between patient and control individuals^47^. In an earlier study, alleles in this gene have been found to linked to family history of schizophrenia^48^. We found a significant interaction between another SNP, rs11846959, located within the SERPINA1 gene and diagnosis. Through analysis stratified by diagnosis, we demonstrated that rs11846959 was significantly associated with expression of the circulating AAT in the control group but not in the schizophrenia group (Table 3, Fig. 2). In the control group, an increase in the number of major alleles of rs11846959 was significantly associated with a decrease in expression of the AAT protein. Finally, vWF polymorphisms have also been implicated in schizophrenia and bipolar disorder but only in studies investigating co-segregation and genetic associations between von Willebrand’s disease and psychotic disorders^49^. We found that a higher number of major alleles of rs12829220 was significantly associated with an increasing level of the vWF in controls (Table 3, Fig. 2). A limitation of our study was that we were restricted to investigation of SNPs located within genes encoding for a selection of proteins included in the commercially available immunoassay panel. This may explain why we were not able to reproduce recent findings from the largest GWAS study^1^. In addition, despite our efforts to minimise effects of confounding factors on protein expression through implementation of strict exclusion criteria, we cannot rule out the effects of some of the key environmental confounders including menstrual cycle and stress^50^.

In conclusion, the data presented here shows that most of the significant SNP and protein associations and SNP x Diagnosis interactions are either directly or indirectly linked to inflammation responses. We found significant associations between SNPs located within genes and their corresponding encoded circulating proteins. Importantly, we demonstrated that significant SNP x Diagnosis interaction was identified for eight serum proteins suggesting that the effect of SNPs on expression of the respective proteins varies with diagnosis.

## Electronic supplementary material

Supplementary Material
