# Supplementary material for: Associations between SNPs and immune-related circulating proteins in schizophrenia
Source: Sci Rep. 2017 Oct 3;7:12586. doi: 10.1038/s41598-017-12986-0 (PMC5626704; doi:10.1038/s41598-017-12986-0)

Associations between SNPs and immune-related circulating proteins in schizophrenia

Running title: SNP and protein association in schizophrenia

Man K. Chan (PhD)[1], Jason D. Cooper (PhD)[1], Stefanie Heilmann-Heimbach (PhD)[2,3], Josef Frank (PhD)[4], Stephanie H. Witt (PhD)[4], Markus M. Nöthen (MD)[2,3], Johann Steiner (MD, PhD)[5], Marcella Rietschel (MD, PhD)[4*], Sabine Bahn (MD, PhD)[1*]

[1]Department of Chemical Engineering and Biotechnology, University of Cambridge, Cambridge, United Kingdom
[2]Institute of Human Genetics, University of Bonn School of Medicine & University Hospital Bonn, Sigmund-Freud-Strasse 25, D-53127 Bonn, Germany
[3]Department of Genomics, Life & Brain Center, University of Bonn, Sigmund-Freud-Strasse 25, D-53127 Bonn, Germany
[4]Department of Genetic Epidemiology in Psychiatry, Central Institute of Mental Health, Faculty of Medicine Mannheim, University of Heidelberg, J5, 65189 Mannheim, Germany
[5]Department of Psychiatry, University of Magdeburg, Germany

***Shared last authorship**

Correspondence to:

Professor Sabine Bahn, Department of Chemical Engineering and Biotechnology, University of Cambridge, Tennis Court Road, Cambridge, CB2 1QT, United Kingdom. Tel: +44 1223 334 151, Fax: +44 1223 334 162, Email: **sb209@cam.ac.uk**

Professor Marcella Rietschel, Department of Genetic Epidemiology in Psychiatry, Central Institute of Mental Health, Faculty of Medicine Mannheim, University of Heidelberg, J5, 65189 Mannheim, Germany. Phone: +49 621 1703 6052, Fax: +40 621 1703 6055. Email: **Marcella.Rietschel@zi-mannheim.de**

Supplementary Material

Supplementary Table 1. Minor allele frequency (MAF) of SNPs

Table summarising the MAF of 632 SNPs found to be located within protein-coding regions of 128 genes that encode for 132 of the measured proteins

CHR	SNP	Minor allele	Major allele	MAF
1	rs2234167	A	G	0.134
1	rs198389	G	A	0.416
1	rs6680254	A	G	0.024
1	rs1041163	G	A	0.154
1	rs3917018	A	G	0.337
1	rs464218	G	A	0.481
1	rs11102972	G	A	0.201
1	rs12037569	A	C	0.181
1	rs10858092	G	A	0.285
1	rs1999713	G	A	0.345
1	rs333968	G	A	0.103
1	rs756325	G	A	0.154
1	rs3006475	C	A	0.126
1	rs7553796	A	C	0.467
1	exm-rs4537545	A	G	0.357
1	rs4537545	A	G	0.355
1	exm-rs4845625	A	G	0.484
1	exm-rs4129267	A	G	0.347
1	rs4240872	G	A	0.287
1	rs2229238	A	G	0.241
1	rs7514452	G	A	0.243
1	rs2765501	A	G	0.447
1	rs16839299	G	A	0.050
1	rs11582594	A	G	0.450
1	rs12074997	A	C	0.117
1	rs7520880	C	A	0.404
1	rs7524550	G	A	0.477
1	rs11264845	G	A	0.351
1	rs3753867	G	A	0.203
1	rs2808661	A	G	0.151
1	exm-rs2794520	A	G	0.350
1	rs2794520	A	G	0.350
1	rs3093077	C	A	0.060
1	exm-rs2808630	G	A	0.256
1	exm-rs3093059	G	A	0.059
1	rs3122012	G	A	0.324
1	rs5082	G	A	0.441
1	rs4786	G	A	0.253
1	rs3917419	A	G	0.444
1	rs10489182	G	A	0.210
1	rs969310	A	G	0.300
1	rs12027035	C	A	0.147
1	rs12135884	G	A	0.289
1	rs7524776	G	A	0.160
1	rs34388368	A	C	0.239
1	rs800292	A	G	0.251
1	exm-rs1329424	A	C	0.386
1	rs572515	A	G	0.383
1	rs10801555	A	G	0.386

1	rs10754199	A	G	0.386
1	exm-rs10737680	C	A	0.431
1	exm-rs6677604	A	G	0.211
1	rs6677604	A	G	0.211
1	exm-rs1410996	A	G	0.434
1	rs1410996	A	G	0.434
1	rs395544	A	G	0.424
1	rs149403976	A	G	0.026
1	exm-rs380390	G	C	0.426
1	rs7540032	A	G	0.433
1	rs2284664	A	G	0.221
1	exm-rs1329428	A	G	0.434
1	rs1065489	A	C	0.137
1	exm-rs3024505	A	G	0.173
1	exm-rs3024493	A	C	0.173
1	exm-rs1518111	A	G	0.194
1	exm-rs1800871	A	G	0.231
1	rs1800871	A	G	0.231
1	rs1800896	A	G	0.490
1	rs2478545	A	G	0.233
1	rs6687360	A	G	0.397
1	rs699	G	A	0.464
1	rs11122576	G	A	0.091
1	rs2071404	A	C	0.130
2	rs1042031	A	G	0.176
2	rs676210	A	G	0.219
2	exm-rs673548	A	G	0.219
2	rs10199768	A	C	0.439
2	rs512535	A	G	0.487
2	rs1713222	A	G	0.136
2	rs4848300	G	A	0.291
2	exm-rs6542095	G	A	0.308
2	rs2856837	A	G	0.289
2	rs17042888	A	G	0.110
2	rs13382561	G	A	0.343
2	rs11677140	C	A	0.294
2	rs1688072	G	A	0.186
2	rs315931	C	A	0.334
2	rs315919	A	C	0.437
2	rs3213448	A	G	0.144
2	rs423904	A	G	0.300
2	rs3087266	A	G	0.166
2	rs315952	G	A	0.319
2	rs9005	A	G	0.318
2	rs1525608	A	G	0.407
2	rs9341105	G	A	0.253
2	rs9341130	G	A	0.092
3	rs6123	G	A	0.447
3	rs8178607	A	G	0.250
3	rs4414894	A	C	0.154
3	rs310001	G	A	0.365
3	rs17241868	G	A	0.394
3	rs73215928	G	A	0.019
3	rs3853148	A	C	0.439
3	rs16839139	A	G	0.037
3	rs9873881	G	A	0.262
3	rs8177191	A	G	0.159
3	exm-rs1799852	A	G	0.101
3	rs6762719	G	A	0.331

3	rs1880669	A	G	0.423
3	exm-rs3811647	A	G	0.328
3	rs8649	G	C	0.247
3	rs3749229	A	G	0.103
3	rs6788635	A	G	0.294
3	rs2070633	A	G	0.480
3	rs13073106	G	A	0.359
3	rs182052	A	G	0.341
3	rs822396	G	A	0.185
3	exm-rs17366568	A	G	0.127
3	rs1501299	A	C	0.287
3	rs3821799	A	G	0.441
3	rs3774262	A	G	0.083
3	exm-rs6773957	A	G	0.373
3	rs2056469	G	A	0.113
3	rs1464504	A	G	0.279
3	rs6605338	G	A	0.264
3	rs4677695	A	G	0.154
4	rs16849364	A	G	0.200
4	rs4640638	A	G	0.207
4	rs2298839	A	G	0.384
4	rs3117598	A	C	0.346
4	rs1429638	A	C	0.204
4	rs2472649	A	G	0.150
4	rs3775488	G	A	0.373
4	rs11727548	G	A	0.357
4	rs4585380	A	G	0.260
4	rs9307118	G	A	0.403
4	rs10009801	G	A	0.161
4	rs1377032	G	A	0.111
4	rs977266	A	G	0.461
4	rs1349634	A	G	0.367
4	rs8878	A	G	0.423
4	rs4256246	A	G	0.163
4	rs12504339	G	A	0.244
4	rs1077234	G	A	0.217
4	rs1596231	G	A	0.351
4	rs355679	A	C	0.170
4	rs189587	A	G	0.420
4	rs355687	G	A	0.239
4	rs920666	G	A	0.236
4	rs142545798	C	A	0.034
4	rs6813526	G	A	0.312
4	rs4754	G	A	0.281
4	rs77630987	A	G	0.011
4	rs7685225	G	A	0.349
4	rs7675246	A	G	0.291
4	rs4444903	G	A	0.397
4	rs79645795	A	G	0.094
4	rs2298982	G	A	0.093
4	rs4698756	A	G	0.307
4	rs9992755	G	A	0.301
4	rs11568943	A	G	0.091
4	rs9991367	A	G	0.093
4	rs11568995	A	G	0.029
4	rs2237051	A	G	0.369
4	rs11569121	A	G	0.079
4	rs17041230	A	G	0.099
4	rs11729130	A	G	0.443

4	rs13147688	G	A	0.193
4	rs1901173	G	A	0.164
4	rs747004	G	A	0.271
4	rs13117654	G	A	0.269
4	rs998310	A	C	0.428
4	rs1519551	G	A	0.394
4	rs2322301	A	G	0.473
4	rs6850492	A	G	0.383
4	rs1493025	G	A	0.474
5	rs739719	A	C	0.081
5	exm-rs1295686	A	G	0.231
5	rs20541	A	G	0.230
5	rs10875633	A	G	0.481
5	rs13385	A	G	0.261
5	rs2237077	G	A	0.286
5	rs4150196	G	A	0.489
5	rs2277025	G	A	0.407
5	rs13173581	A	G	0.271
5	rs2279804	A	G	0.476
5	rs6873777	A	G	0.387
5	exm-rs3213094	A	G	0.166
5	rs2569254	A	G	0.174
5	rs3212220	A	C	0.166
5	exm-rs2546890	G	A	0.447
6	rs4960348	G	A	0.331
6	exm-rs3812163	A	T	0.461
6	rs1107495	G	A	0.164
6	rs2068361	A	G	0.264
6	rs12214906	C	A	0.460
6	rs9505270	A	G	0.307
6	rs270404	G	A	0.450
6	rs270378	A	G	0.284
6	rs270398	A	C	0.186
6	rs9328444	A	G	0.276
6	rs9379137	G	A	0.353
6	rs13216391	G	A	0.183
6	rs911751	A	C	0.471
6	rs2326994	G	A	0.264
6	rs267802	A	C	0.291
6	rs10498673	A	G	0.114
6	rs267186	A	G	0.395
6	rs6940057	A	G	0.043
6	rs267187	G	A	0.373
6	rs267205	G	A	0.239
6	rs13213419	A	C	0.019
6	rs1225930	A	G	0.277
6	rs1225934	C	A	0.387
6	rs849874	G	A	0.163
6	rs849886	A	G	0.484
6	rs78697234	G	A	0.020
6	exm-rs2009658	G	C	0.166
6	exm-rs2516312	G	A	0.023
6	exm-rs909253	G	A	0.322
6	rs1041981	A	C	0.324
6	exm-rs1799964	G	A	0.211
6	rs1799964	G	A	0.210
6	exm-rs1800630	A	C	0.166
6	exm-rs1800629	A	G	0.176
6	exm-rs361525	A	G	0.041

6	exm-rs3093662	G	A	0.051
6	exm-rs769177	A	G	0.031
6	exm-rs3134947	A	G	0.213
6	exm-rs2269423	A	C	0.367
6	exm-rs3134946	C	G	0.214
6	exm-rs3130349	A	G	0.186
6	exm-rs3134943	A	G	0.137
6	exm-rs1035798	A	G	0.279
6	exm-rs204995	G	A	0.224
6	exm-rs204994	A	G	0.211
6	exm-rs204993	G	A	0.249
6	exm-rs2071292	G	A	0.031
6	exm-rs2022059	G	C	0.036
6	exm-rs204992	A	G	0.209
6	rs833069	G	A	0.299
6	rs833070	G	A	0.484
6	rs3025007	A	G	0.446
6	rs3025035	A	G	0.080
6	exm-rs998584	A	C	0.467
6	rs6900017	A	G	0.091
6	exm-rs6905288	G	A	0.437
6	rs4715332	C	A	0.431
6	rs9342104	A	G	0.444
6	rs2031365	A	G	0.314
6	rs981087	A	G	0.478
6	exm-rs6919346	A	G	0.171
6	rs9365171	A	C	0.354
6	rs10455872	G	A	0.069
6	rs73596816	A	G	0.047
6	exm-rs7770628	G	A	0.451
6	rs6926458	G	A	0.257
6	rs7761377	G	A	0.376
6	exm-rs1652507	G	A	0.176
6	rs1367211	A	G	0.277
6	rs9346833	G	A	0.450
6	rs783149	A	C	0.179
6	exm-rs1084651	A	G	0.179
7	rs6969537	A	G	0.139
7	rs11770506	G	A	0.337
7	rs763317	A	G	0.484
7	rs11773818	A	G	0.257
7	rs759167	A	C	0.216
7	rs729969	A	G	0.116
7	rs139165969	A	C	0.013
7	rs17172438	G	A	0.164
7	exm-rs11979158	G	A	0.156
7	rs4947490	A	G	0.320
7	rs917880	A	G	0.459
7	rs2110290	G	A	0.351
7	rs13244925	A	C	0.456
7	rs117479531	G	A	0.010
7	rs17172446	A	G	0.253
7	rs6964705	A	C	0.439
7	rs2072454	G	A	0.450
7	rs10228436	A	G	0.337
7	rs2017000	G	A	0.274
7	rs9692301	G	A	0.287
7	rs7795743	G	A	0.420
7	rs6593211	A	G	0.231

7	rs1107617	C	A	0.073
7	rs1111650	G	A	0.384
7	rs7776830	C	A	0.101
7	rs10233099	A	G	0.056
7	rs12718947	A	G	0.307
7	rs12536061	G	A	0.356
7	rs17746319	G	A	0.139
7	rs2037701	A	G	0.454
7	rs6948867	A	G	0.207
7	rs6467865	A	G	0.187
7	rs12540393	G	A	0.209
7	rs1734908	C	A	0.437
7	rs1734907	A	G	0.166
7	rs6950982	G	A	0.191
7	rs7242	C	A	0.460
7	rs1050955	A	G	0.200
7	rs12706832	A	G	0.483
7	rs28954099	A	G	0.027
7	rs11760956	A	G	0.394
7	rs2060715	A	G	0.479
8	rs2442474	G	A	0.026
8	rs2167071	G	A	0.376
8	rs2515409	G	A	0.138
8	rs2442631	A	G	0.451
8	rs2916747	G	A	0.046
8	rs2515432	A	G	0.497
8	rs1961222	A	G	0.324
8	rs3824310	G	A	0.317
8	rs2442608	G	A	0.470
8	rs734704	A	G	0.269
8	rs2515481	G	A	0.403
8	rs2959812	A	G	0.269
8	rs2897911	C	A	0.335
8	rs2922873	A	G	0.144
8	rs75915835	G	A	0.177
8	rs2922894	G	A	0.011
8	rs3739391	A	G	0.129
8	rs12545375	G	A	0.034
8	rs7830593	A	G	0.196
8	rs74820521	G	A	0.021
8	rs140965086	A	C	0.017
8	rs12681965	C	A	0.205
8	rs11779793	A	C	0.086
8	rs181323638	A	G	0.021
8	rs12550612	G	A	0.199
8	rs17057438	A	G	0.303
8	exm-rs17466684	A	G	0.186
8	rs10503814	A	G	0.030
8	exm-rs2279590	A	G	0.397
8	rs9331931	G	C	0.256
8	exm-rs11136000	A	G	0.393
8	rs11136000	A	G	0.391
8	exm-rs1532278	A	G	0.381
8	rs34109053	A	G	0.266
8	rs538181	G	A	0.431
8	rs17504422	G	A	0.040
8	rs10957898	A	G	0.236
8	rs2717540	A	G	0.189
8	rs16906018	G	A	0.039

8	rs1026920	G	A	0.266
8	rs6993386	G	A	0.360
8	rs2717548	A	G	0.186
8	rs2717537	G	A	0.189
8	exm-rs2717536	A	G	0.116
9	rs10759680	A	G	0.224
9	rs2285055	G	A	0.363
9	rs12347433	G	A	0.246
9	rs953288	C	A	0.386
9	rs10122770	A	G	0.046
9	rs1547691	A	G	0.264
9	rs11794797	A	G	0.277
9	rs1330368	A	G	0.469
9	rs7847271	A	G	0.093
9	rs7043308	G	A	0.181
9	rs11791562	A	G	0.179
9	rs117778560	G	A	0.031
9	rs944510	A	G	0.417
9	rs1330360	A	G	0.367
9	rs10982536	C	A	0.149
9	rs2071520	G	A	0.322
9	rs3814526	G	A	0.046
10	rs4934434	A	C	0.426
10	rs1800682	G	A	0.433
10	rs4406737	A	G	0.397
10	exm-rs2234978	A	G	0.282
10	rs2234978	A	G	0.284
10	rs1977389	C	A	0.422
10	rs1468063	A	G	0.074
10	rs2234971	A	G	0.110
10	rs11190087	A	G	0.421
11	rs3842752	A	G	0.190
11	rs1519480	G	A	0.290
11	rs6265	A	G	0.200
11	rs10835210	A	C	0.443
11	rs10835211	A	G	0.234
11	rs34043390	G	A	0.016
11	rs117853517	A	G	0.019
11	exm-rs10767664	T	A	0.221
11	rs2030323	A	C	0.220
11	rs7934165	G	A	0.491
11	rs6169	A	G	0.426
11	rs1109748	A	C	0.081
11	rs760306	A	G	0.307
11	rs3758977	C	A	0.343
11	rs2156528	A	C	0.274
11	rs1996352	G	A	0.211
11	rs7948454	G	A	0.086
11	rs470168	A	G	0.324
11	rs4431992	G	A	0.227
11	rs2276108	G	A	0.121
11	rs4754880	A	G	0.193
11	rs2071230	G	A	0.102
11	rs2408490	A	G	0.174
11	rs566125	A	G	0.153
11	rs679620	A	G	0.476
11	rs522616	G	A	0.208
11	rs544354	A	G	0.120
11	rs5744256	G	A	0.299

11	exm-rs1834481	C	G	0.299
11	rs360717	A	G	0.287
11	rs2849176	A	G	0.424
11	rs2070665	A	G	0.079
12	rs3809241	C	A	0.142
12	rs2286646	G	A	0.217
12	rs12317523	A	G	0.262
12	rs12829220	A	G	0.030
12	rs917859	A	G	0.386
12	rs216865	A	G	0.337
12	rs216873	A	G	0.197
12	rs216888	A	G	0.446
12	rs3741906	A	G	0.089
12	rs216800	A	G	0.320
12	rs11063996	G	A	0.237
12	rs11608550	A	G	0.237
12	rs216334	G	A	0.344
12	rs216291	G	A	0.443
12	rs1063856	G	A	0.330
12	rs140164271	A	G	0.033
12	rs980131	A	G	0.379
12	rs7139057	C	A	0.170
12	rs12319392	A	C	0.101
12	rs7135976	G	A	0.494
12	rs116856265	G	A	0.039
12	rs3819537	G	A	0.327
12	rs2109118	A	G	0.469
12	rs2239144	A	C	0.113
12	rs11064024	G	A	0.386
12	rs11829713	A	G	0.064
12	rs2286608	G	A	0.396
12	rs3026229	A	C	0.163
12	rs7955940	A	G	0.357
12	rs1805659	G	A	0.358
12	exm2221241	G	A	0.277
12	rs4882978	G	A	0.172
12	rs226381	G	A	0.474
12	rs11053646	C	G	0.082
12	rs2742115	G	A	0.250
12	rs2742113	A	C	0.311
12	rs7315498	G	A	0.387
12	exm-rs995030	A	G	0.200
12	rs1000788	G	A	0.210
12	exm-rs3782181	C	A	0.211
12	exm-rs4474514	G	A	0.211
12	rs3907470	G	A	0.210
12	exm-rs5742692	G	A	0.014
12	rs978458	A	G	0.270
12	rs5742671	A	G	0.186
12	rs4764884	A	G	0.257
12	rs5742632	G	A	0.240
12	exm-rs35767	A	G	0.153
13	rs555212	A	G	0.238
13	rs561241	G	A	0.105
13	rs6041	A	G	0.099
13	rs3212994	A	G	0.034
13	rs3211719	G	A	0.257
14	rs3759614	G	A	0.170
14	rs10137082	A	G	0.223

14	rs3811178	A	G	0.299
14	rs723840	A	G	0.411
14	rs72734245	A	G	0.033
14	rs2268613	G	A	0.179
14	rs9658634	A	G	0.240
14	rs9658644	A	G	0.263
14	rs2295396	G	A	0.238
14	rs729940	A	G	0.143
14	rs750678	G	A	0.336
14	rs2281514	A	G	0.477
14	rs11846959	A	G	0.214
14	rs6647	G	A	0.221
14	rs20546	A	G	0.020
14	rs1980616	A	G	0.204
14	rs1884082	C	A	0.476
14	rs8007632	A	G	0.226
14	rs2071407	A	G	0.364
15	rs2169830	G	A	0.293
15	rs76698147	G	A	0.031
15	rs7169204	G	A	0.214
15	rs17336789	A	G	0.177
15	rs7176848	G	A	0.279
15	rs117196289	C	A	0.019
15	rs4451910	A	G	0.091
15	rs925272	A	G	0.030
15	rs1995830	G	A	0.215
15	rs12437819	A	C	0.103
15	rs12907134	G	A	0.439
15	rs7179134	A	G	0.206
15	exm-rs7172689	A	G	0.206
15	rs4589514	G	A	0.126
15	rs8031107	A	G	0.444
15	rs11857713	A	G	0.074
15	rs11073001	G	A	0.196
15	rs17875563	T	A	0.233
15	rs4778641	G	A	0.376
15	rs4617815	G	A	0.187
16	exm-rs12444268	A	T	0.301
16	rs11859916	A	G	0.123
16	rs9646256	A	G	0.357
16	rs11647727	A	G	0.226
16	exm-rs4293393	G	A	0.163
16	rs4293393	G	A	0.163
16	exm-rs13333226	G	A	0.165
16	exm-rs12917707	A	C	0.159
16	rs9652589	A	G	0.494
16	rs4490010	A	G	0.026
16	rs223819	G	A	0.070
16	rs223812	G	A	0.270
16	rs13338993	G	A	0.059
16	rs2070937	A	G	0.479
17	rs12150660	A	C	0.230
17	rs2541012	A	G	0.419
17	rs858520	A	G	0.403
17	rs727428	A	G	0.400
17	rs2277667	G	A	0.084
17	rs2227725	A	G	0.034
17	rs991804	A	G	0.270
17	rs4795895	A	G	0.184

17	rs17735961	A	C	0.183
17	rs17809012	G	A	0.476
17	rs1431991	G	A	0.456
17	rs3136674	G	A	0.040
17	rs2282692	C	A	0.366
17	rs1065341	G	A	0.076
17	rs2063979	G	A	0.299
17	rs1719200	G	A	0.085
17	rs860559	G	A	0.227
17	rs76020764	G	A	0.017
17	rs854477	A	G	0.331
17	rs1851503	C	A	0.379
17	rs1719126	G	A	0.260
17	rs9972960	A	G	0.393
17	rs8078723	G	A	0.384
17	rs2827	A	G	0.179
17	rs12453732	C	A	0.439
17	rs2302776	G	A	0.443
17	rs231471	G	A	0.444
17	rs12949848	G	A	0.130
17	rs4329	G	A	0.483
17	rs4331	G	A	0.481
17	exm-rs4343	A	G	0.490
17	rs12709437	A	G	0.029
17	rs4362	G	A	0.490
17	rs3020619	G	A	0.255
17	rs8178869	C	A	0.158
17	rs4791079	A	C	0.419
17	rs8178855	C	A	0.340
17	rs9892748	C	A	0.241
17	rs8079985	G	A	0.104
17	rs117534989	A	G	0.024
17	rs8066294	G	A	0.224
17	rs2215413	C	A	0.423
18	rs1800458	A	G	0.093
18	rs3794884	C	A	0.321
19	rs11569562	A	G	0.486
19	rs344541	G	A	0.364
19	rs11569523	A	G	0.070
19	rs423490	A	G	0.254
19	rs428453	G	C	0.388
19	rs11672613	G	A	0.425
19	rs4807895	A	G	0.340
19	rs2230199	C	G	0.200
19	rs339392	C	A	0.224
19	rs79681647	C	A	0.039
19	rs4804765	A	C	0.321
19	rs80035917	A	C	0.067
19	rs3136653	A	G	0.194
19	rs3136657	G	A	0.371
19	rs281440	G	A	0.266
19	rs1799969	A	G	0.143
19	rs5498	G	A	0.426
19	exm-rs281437	A	G	0.240
19	exm-rs3093030	A	G	0.417
19	rs16975079	G	A	0.020
19	rs8101017	A	C	0.284
19	rs2301236	A	G	0.195
19	rs3760659	C	A	0.264

19	rs740591	A	G	0.037
19	rs1076362	A	G	0.146
19	rs1029804	C	A	0.203
19	rs4090570	G	A	0.318
19	rs2215324	A	G	0.347
19	rs1990301	A	C	0.213
19	rs2109075	A	G	0.463
19	rs11083635	A	G	0.293
19	rs9304597	A	G	0.250
19	rs1860329	G	A	0.467
19	rs10407999	A	C	0.241
19	rs7251044	A	G	0.019
19	rs7256597	C	A	0.193
19	rs1593086	A	G	0.313
19	rs7249230	A	G	0.174
19	exm-rs769449	A	G	0.130
19	rs769449	A	G	0.130
19	rs7412	A	G	0.096
19	rs72654473	A	C	0.110
19	exm-rs439401	A	G	0.384
19	exm-rs445925	A	G	0.110
19	rs445925	A	G	0.110
19	rs7256200	A	C	0.128
19	exm-rs4420638	G	A	0.186
19	rs4420638	G	A	0.186
19	snv-rs141622900	A	G	0.069
20	rs3176123	C	A	0.192
20	exm-rs911119	G	A	0.223
20	rs2424577	G	A	0.404
20	rs3827143	G	A	0.216
20	rs3918240	A	G	0.352
20	rs3918281	A	G	0.034
20	rs2250889	C	G	0.050
20	rs13925	A	G	0.129
21	rs1041740	A	G	0.266
22	rs875643	A	G	0.384
22	rs738806	A	G	0.269
22	rs5751759	G	A	0.191
22	rs1007888	G	A	0.434
22	rs17004049	G	A	0.063
22	rs4821423	A	G	0.386
22	rs5755790	G	A	0.440
22	rs7293	A	G	0.459
22	rs17722827	A	G	0.061
22	rs5750141	G	A	0.469
22	rs9611117	C	A	0.429
22	rs5750781	A	C	0.273
23	rs6609533	G	A	0.497
23	rs6520278	A	G	0.388
23	rs4824621	G	A	0.352
23	rs1804495	A	C	0.080
23	rs1126535	G	A	0.178

Supplementary Table 2. Table listing all the significant SNP and protein expression associations stratified by diagnosis

						Controls			Schizophrenia		
**Protein**	**Protein Abbrev**	**Gene**	**SNP**	**Chr**	**Interaction P-value**	**β**	**P-value**	**Adjusted P-value**	**β**	**P-value**	**Adjusted P-value**
**Interleukin-6 receptor**	IL-6r	IL6R	**rs7553796**	1	3.88E-02	0.28	5.64E-08	9.44E-07	0.43	5.23E-12	3.33E-10
**Coagulation factor VII**	Factor VII	F7	**rs555212**	13	1.52E-02	-0.30	2.03E-06	3.07E-05	-0.04	5.21E-01	8.31E-01
**Alpha-1-antitrypsin**	AAT	SERPINA1	**rs11846959**	14	3.72E-04	-0.19	3.29E-04	3.81E-03	0.08	5.16E-02	3.22E-01
**CXCL10(1-73)**	IP-10	CXCL10	**rs4256246**	4	9.85E-03	0.23	4.38E-03	3.77E-02	-0.21	9.58E-02	4.41E-01
**von Willebrand factor**	vWF	VWF	**rs12829220**	12	1.01E-02	0.75	5.79E-03	4.72E-02	-0.23	3.83E-01	7.45E-01
**Tenascin**	TN C	TNC	**rs7043308**	9	1.69E-02	0.19	1.09E-02	7.85E-02	-0.09	3.12E-01	6.89E-01
**Vasostatin-1**	CgA	CHGA	**rs2295396**	14	2.40E-03	0.53	1.61E-02	1.05E-01	-0.54	2.74E-02	2.10E-01
**E-selectin**	E Selectin	SELE	**rs3917419**	1	7.98E-03	-0.11	3.20E-02	1.77E-01	0.13	1.37E-01	5.33E-01
**Leptin**	Leptin	LEP	**rs28954099**	7	5.27E-03	-0.60	4.05E-02	2.05E-01	0.75	8.02E-02	3.98E-01
**Vasostatin-1**	CgA	CHGA	**rs9658644**	14	2.45E-03	-0.13	5.29E-01	7.90E-01	0.77	6.43E-04	7.71E-03
**Cystatin-C**	Cystatin C	CST3	**rs2424577**	20	3.32E-02	0.02	4.63E-01	7.50E-01	0.09	7.54E-04	8.88E-03
**Vitamin K-dependent protein S**	VKDPS	PROS1	**rs6123**	3	2.30E-02	-0.01	8.09E-01	9.32E-01	0.09	3.24E-03	3.38E-02
**Vasostatin-1**	CgA	CHGA	**rs750678**	14	1.39E-02	-0.13	4.89E-01	7.56E-01	0.57	1.10E-02	1.04E-01
**Interleukin-12 subunit beta**	IL-12p40	IL12B	**rs2569254**	5	1.23E-02	0.12	2.65E-01	5.69E-01	-0.29	1.44E-02	1.27E-01
**C-X-C motif chemokine 13**	BLC	CXCL13	**rs142545798**	4	2.86E-02	-0.32	5.63E-01	8.15E-01	1.60	1.57E-02	1.35E-01
**CD5 antigen-like**	CD5L	CD5L	**rs16839299**	1	4.73E-02	0.07	4.35E-01	7.42E-01	-0.35	3.86E-02	2.64E-01
**Matrix metalloproteinase-9**	MMP-9 total	MMP9	**rs13925**	20	1.35E-02	-0.08	3.63E-01	6.84E-01	0.18	5.32E-02	3.28E-01
**Resistin**	Resistin	RETN	**rs80035917**	19	2.92E-02	-0.12	1.93E-01	4.94E-01	0.11	2.61E-01	6.56E-01
**Epidermal growth factor receptor**	EGFR	EGFR	**rs2072454**	7	3.53E-02	-0.03	1.83E-01	4.87E-01	0.03	3.48E-01	7.18E-01
**Soluble KIT ligand**	SCF	KITLG	**exm-rs995030**	12	4.48E-02	-0.12	7.20E-02	2.94E-01	0.07	3.71E-01	7.37E-01
**Prolactin**	PRL	PRL	**rs78697234**	6	3.26E-02	-0.66	1.18E-01	3.87E-01	0.20	7.27E-01	9.11E-01
**22 kDa interstitial collagenase**	MMP-1	MMP1	**rs2071230**	11	2.78E-01	-0.42	3.70E-03	3.41E-02	-0.19	3.22E-01	6.97E-01
**22 kDa interstitial collagenase**	MMP-1	MMP1	**rs2408490**	11	3.17E-01	0.30	2.00E-02	1.26E-01	0.11	4.12E-01	7.60E-01
**Adiponectin**	Adiponectin	ADIPOQ	**exm-rs17366568**	3	1.79E-01	0.33	8.09E-04	9.03E-03	0.06	6.24E-01	8.89E-01
**Advanced glycosylation end product-specific receptor**	RAGE	AGER	**exm-rs2022059**	6	5.46E-02	0.94	1.10E-05	1.55E-04	0.16	5.24E-01	8.33E-01
**Advanced glycosylation end product-specific receptor**	RAGE	AGER	**exm-rs204993**	6	2.88E-01	0.20	1.43E-02	9.37E-02	0.03	7.74E-01	9.31E-01
**Advanced glycosylation end product-specific receptor**	RAGE	AGER	**exm-rs2269423**	6	8.30E-01	-0.15	3.24E-02	1.77E-01	-0.19	5.56E-02	3.34E-01
**Alpha-1-antitrypsin**	AAT	SERPINA1	**rs6647**	14	2.38E-01	0.10	4.69E-02	2.19E-01	0.01	8.15E-01	9.46E-01
**Alpha-2-HS-glycoprotein chain B**	Fetuin A	AHSG	**rs6788635**	3	9.62E-01	0.22	3.33E-11	8.14E-10	0.23	2.30E-06	4.56E-05
**Alpha-2-HS-glycoprotein chain B**	Fetuin A	AHSG	**rs2070633**	3	2.11E-01	0.21	4.56E-11	1.08E-09	0.27	1.11E-11	5.88E-10
**Alpha-2-HS-glycoprotein chain B**	Fetuin A	AHSG	**rs13073106**	3	4.27E-01	0.20	3.37E-10	6.30E-09	0.26	3.64E-09	1.01E-07
**Alpha-2-macroglobulin**	A2Macro	A2M	**rs3026229**	12	2.93E-01	-0.03	4.62E-01	7.50E-01	-0.14	9.15E-03	8.82E-02
**Alpha-2-macroglobulin**	A2Macro	A2M	**rs4882978**	12	4.00E-01	-0.05	2.76E-01	5.80E-01	-0.13	1.13E-02	1.05E-01
**Angiopoietin-2**	ANG-2	ANGPT2	**rs3739391**	8	1.28E-01	-0.31	2.89E-02	1.70E-01	0.14	4.21E-01	7.61E-01
**Angiopoietin-2**	ANG-2	ANGPT2	**rs2442474**	8	7.65E-01	-0.53	4.97E-02	2.31E-01	-0.46	2.39E-01	6.45E-01
**Angiotensin-4**	Angiotensinogen	AGT	**rs2478545**	1	5.21E-01	-1.78	2.73E-16	1.45E-14	-1.97	1.14E-08	2.91E-07
**Angiotensin-4**	Angiotensinogen	AGT	**rs6687360**	1	8.65E-01	-1.42	6.09E-12	1.71E-10	-1.44	1.40E-05	2.49E-04
**Angiotensin-4**	Angiotensinogen	AGT	**rs699**	1	6.16E-01	-1.30	5.78E-11	1.31E-09	-1.42	1.54E-05	2.64E-04
**Angiotensin-4**	Angiotensinogen	AGT	**rs2071404**	1	8.71E-02	-0.06	8.61E-01	9.51E-01	0.94	4.62E-02	3.00E-01
**Angiotensin-converting enzyme**	ACE	ACE	**exm-rs4343**	17	8.02E-01	0.41	4.97E-18	3.95E-16	0.37	1.33E-10	4.44E-09
**Angiotensin-converting enzyme**	ACE	ACE	**rs4362**	17	9.51E-01	0.41	7.42E-18	5.24E-16	0.38	1.22E-11	5.97E-10
**Angiotensin-converting enzyme**	ACE	ACE	**rs4331**	17	8.68E-01	0.40	2.26E-17	1.44E-15	0.36	2.53E-10	7.65E-09
**Angiotensin-converting enzyme**	ACE	ACE	**rs4329**	17	8.81E-01	0.40	4.24E-17	2.45E-15	0.36	2.53E-10	7.65E-09
**Angiotensin-converting enzyme**	ACE	ACE	**rs12709437**	17	8.94E-02	0.00	9.86E-01	9.99E-01	-0.36	1.20E-02	1.09E-01

**Apolipoprotein A-IV**	Apo-A-IV	APOA4	**rs2849176**	11	4.05E-01	0.16	8.45E-03	6.55E-02	0.02	7.81E-01	9.32E-01
**Apolipoprotein B-48**	Apo-B	APOB	**rs1713222**	2	5.10E-02	-0.03	6.52E-01	8.58E-01	0.17	1.60E-02	1.35E-01
**Apolipoprotein C-I**	Apo-C-I	APOC1	**snv-rs141622900**	19	2.83E-01	-0.20	1.67E-02	1.06E-01	-0.06	5.07E-01	8.20E-01
**Apolipoprotein C-I**	Apo-C-I	APOC1	**rs7256200**	19	1.45E-01	-0.03	6.40E-01	8.53E-01	-0.14	2.62E-02	2.05E-01
**Apolipoprotein C-I**	Apo-C-I	APOC1	**exm-rs4420638**	19	3.61E-01	-0.07	1.95E-01	4.94E-01	-0.12	3.69E-02	2.55E-01
**Apolipoprotein C-I**	Apo-C-I	APOC1	**rs4420638**	19	3.61E-01	-0.07	1.95E-01	4.94E-01	-0.12	3.69E-02	2.55E-01
**Apolipoprotein E**	Apo-E	APOE	**rs7412**	19	3.81E-01	-0.49	1.08E-05	1.55E-04	-0.63	2.25E-08	5.10E-07
**Apolipoprotein E**	Apo-E	APOE	**rs72654473**	19	2.55E-01	-0.35	1.08E-03	1.16E-02	-0.51	9.79E-07	2.08E-05
**Apolipoprotein E**	Apo-E	APOE	**exm-rs769449**	19	5.21E-02	0.27	4.18E-03	3.69E-02	0.53	2.22E-08	5.10E-07
**Apolipoprotein E**	Apo-E	APOE	**rs769449**	19	5.21E-02	0.27	4.18E-03	3.69E-02	0.53	2.22E-08	5.10E-07
**Apolipoprotein E**	Apo-E	APOE	**exm-rs439401**	19	9.15E-01	0.15	2.02E-02	1.26E-01	0.11	1.22E-01	4.99E-01
**Apolipoprotein(a)**	Lpa	LPA	**rs10455872**	6	7.57E-02	-3.01	4.14E-12	1.25E-10	-2.10	2.50E-09	7.21E-08
**Apolipoprotein(a)**	Lpa	LPA	**rs73596816**	6	9.49E-01	-2.16	5.72E-06	8.45E-05	-2.05	6.04E-04	7.39E-03
**Apolipoprotein(a)**	Lpa	LPA	**rs6926458**	6	3.21E-01	0.91	5.44E-05	6.92E-04	0.70	3.16E-03	3.35E-02
**Apolipoprotein(a)**	Lpa	LPA	**exm-rs7770628**	6	9.12E-01	-0.74	3.46E-04	3.93E-03	-0.86	5.41E-05	7.65E-04
**Apolipoprotein(a)**	Lpa	LPA	**rs7761377**	6	9.09E-01	0.53	9.71E-03	7.18E-02	0.60	6.16E-03	6.12E-02
**Apolipoprotein(a)**	Lpa	LPA	**rs9365171**	6	6.12E-01	0.53	1.16E-02	8.10E-02	0.41	5.94E-02	3.47E-01
**Apolipoprotein(a)**	Lpa	LPA	**rs9346833**	6	4.08E-01	0.45	3.06E-02	1.77E-01	0.64	2.35E-03	2.58E-02
**Beta-2-glycoprotein 1**	Apo-H	APOH	**rs9892748**	17	5.33E-01	-0.09	4.23E-02	2.07E-01	-0.06	2.94E-01	6.79E-01
**BNP(4-29)**	BNP	NPPB	**rs198389**	1	8.13E-02	-0.20	1.05E-01	3.70E-01	-0.62	9.54E-05	1.32E-03
**Bone morphogenetic protein 6**	BMP-6	BMP6	**rs270398**	6	2.81E-01	0.39	2.53E-03	2.44E-02	0.18	3.72E-01	7.37E-01
**Bone morphogenetic protein 6**	BMP-6	BMP6	**rs911751**	6	6.80E-01	0.31	2.86E-03	2.71E-02	0.30	6.44E-02	3.62E-01
**Bone morphogenetic protein 6**	BMP-6	BMP6	**rs270378**	6	1.88E-01	0.29	1.12E-02	7.89E-02	0.07	7.15E-01	9.11E-01
**Bone morphogenetic protein 6**	BMP-6	BMP6	**rs1107495**	6	7.02E-01	-0.35	1.17E-02	8.10E-02	-0.21	3.11E-01	6.89E-01
**Bone morphogenetic protein 6**	BMP-6	BMP6	**rs267187**	6	1.66E-01	-0.27	1.30E-02	8.91E-02	0.06	7.35E-01	9.11E-01
**Bone morphogenetic protein 6**	BMP-6	BMP6	**rs267186**	6	2.66E-01	-0.24	3.15E-02	1.77E-01	0.03	8.36E-01	9.56E-01
**Bone morphogenetic protein 6**	BMP-6	BMP6	**rs13216391**	6	7.89E-01	-0.27	6.00E-02	2.62E-01	-0.46	1.73E-02	1.43E-01
**Carcinoembryonic antigen-related cell adhesion molecule 5**	CEA	CEACAM5	**rs9304597**	19	6.92E-02	-0.33	2.81E-02	1.69E-01	0.11	5.09E-01	8.20E-01
**Carcinoembryonic antigen-related cell adhesion molecule 5**	CEA	CEACAM5	**rs10407999**	19	1.83E-01	0.31	3.50E-02	1.88E-01	0.00	9.87E-01	9.94E-01
**C-C motif chemokine 16**	HCC 4	CCL16	**rs2063979**	17	4.00E-01	0.23	4.18E-03	3.69E-02	0.09	2.39E-01	6.45E-01
**C-C motif chemokine 18**	PARC	CCL18	**rs854477**	17	8.72E-01	0.16	1.34E-02	9.09E-02	0.13	6.11E-02	3.50E-01
**C-C motif chemokine 23**	MPIF 1	CCL23	**rs1719200**	17	4.97E-01	0.23	5.25E-03	4.40E-02	0.14	9.97E-02	4.47E-01
**C-C motif chemokine 23**	MPIF 1	CCL23	**rs860559**	17	5.58E-01	0.15	8.25E-03	6.48E-02	0.20	2.31E-03	2.57E-02
**CD40 ligand**	CD40 L	CD40LG	**rs1126535**	23	6.11E-01	-0.25	4.26E-02	2.07E-01	-0.13	2.73E-01	6.62E-01
**CD5 antigen-like**	CD5L	CD5L	**rs2765501**	1	7.54E-01	-0.15	3.30E-04	3.81E-03	-0.17	8.41E-04	9.73E-03
**Coagulation factor VII**	Factor VII	F7	**rs561241**	13	3.41E-01	0.37	2.26E-05	3.05E-04	0.44	8.31E-09	2.20E-07
**Coagulation factor VII**	Factor VII	F7	**rs6041**	13	9.28E-01	0.37	4.74E-05	6.15E-04	0.39	2.21E-06	4.53E-05
**Complement C3 alpha chain**	C3	C3	**rs11569562**	19	4.78E-01	0.04	1.72E-01	4.78E-01	0.07	2.54E-02	2.02E-01
**Complement C3 alpha chain**	C3	C3	**rs79681647**	19	1.97E-01	0.05	4.79E-01	7.50E-01	0.20	3.22E-02	2.33E-01
**Complement factor H**	CFH	CFH	**exm-rs6677604**	1	1.55E-01	0.76	1.10E-61	3.51E-59	0.67	1.03E-26	3.26E-24
**Complement factor H**	CFH	CFH	**rs6677604**	1	1.55E-01	0.76	1.10E-61	3.51E-59	0.67	1.03E-26	3.26E-24
**Complement factor H**	CFH	CFH	**exm-rs10737680**	1	1.02E-01	0.49	1.13E-21	1.43E-19	0.38	1.29E-10	4.44E-09
**Complement factor H**	CFH	CFH	**exm-rs1329428**	1	1.48E-01	0.49	1.13E-21	1.43E-19	0.39	3.01E-11	1.13E-09
**Complement factor H**	CFH	CFH	**rs7540032**	1	1.48E-01	0.49	1.13E-21	1.43E-19	0.39	3.01E-11	1.13E-09
**Complement factor H**	CFH	CFH	**exm-rs1410996**	1	1.39E-01	0.49	1.80E-21	1.63E-19	0.39	3.01E-11	1.13E-09
**Complement factor H**	CFH	CFH	**rs1410996**	1	1.39E-01	0.49	1.80E-21	1.63E-19	0.39	3.01E-11	1.13E-09
**Complement factor H**	CFH	CFH	**exm-rs380390**	1	1.66E-01	-0.35	6.44E-12	1.71E-10	-0.25	1.41E-05	2.49E-04
**Complement factor H**	CFH	CFH	**rs395544**	1	1.69E-01	-0.35	6.44E-12	1.71E-10	-0.26	1.07E-05	2.06E-04
**Complement factor H**	CFH	CFH	**exm-rs1329424**	1	1.70E-01	-0.35	6.82E-11	1.36E-09	-0.25	2.09E-05	3.17E-04

**Complement factor H**	CFH	CFH	**rs10754199**	1	1.70E-01	-0.35	6.82E-11	1.36E-09	-0.25	2.09E-05	3.17E-04
**Complement factor H**	CFH	CFH	**rs10801555**	1	1.70E-01	-0.35	6.82E-11	1.36E-09	-0.25	2.09E-05	3.17E-04
**Complement factor H**	CFH	CFH	**rs572515**	1	1.70E-01	-0.35	6.82E-11	1.36E-09	-0.25	2.09E-05	3.17E-04
**Complement factor H**	CFH	CFH	**rs1065489**	1	4.51E-01	-0.22	9.99E-03	7.31E-02	-0.17	4.00E-02	2.70E-01
**C-reactive protein**	CRP	CRP	**exm-rs2794520**	1	8.57E-01	0.40	4.13E-02	2.05E-01	0.30	2.40E-01	6.45E-01
**C-reactive protein**	CRP	CRP	**rs2794520**	1	8.57E-01	0.40	4.13E-02	2.05E-01	0.30	2.40E-01	6.45E-01
**Creatine kinase B-type**	CK MB	CKB	**rs2071407**	14	1.32E-01	0.05	5.89E-01	8.37E-01	0.30	4.94E-02	3.11E-01
**C-X-C motif chemokine 13**	BLC	CXCL13	**rs1596231**	4	5.89E-01	0.39	4.10E-02	2.05E-01	0.25	3.12E-01	6.89E-01
**C-X-C motif chemokine 5**	ENA 78	CXCL5	**rs2472649**	4	5.17E-01	-0.62	3.15E-11	8.01E-10	-0.54	1.84E-05	3.08E-04
**C-X-C motif chemokine 5**	ENA 78	CXCL5	**rs3775488**	4	2.61E-01	0.18	1.64E-02	1.05E-01	0.05	5.80E-01	8.67E-01
**CXCL10(1-73)**	IP-10	CXCL10	**rs12504339**	4	7.64E-02	0.19	9.03E-03	6.76E-02	-0.07	5.08E-01	8.20E-01
**Cystatin-C**	Cystatin C	CST3	**rs3827143**	20	3.30E-01	0.09	3.30E-03	3.09E-02	0.13	3.20E-04	3.99E-03
**Cystatin-C**	Cystatin C	CST3	**exm-rs911119**	20	1.84E-01	0.08	5.76E-03	4.72E-02	0.14	1.22E-04	1.65E-03
**Epidermal growth factor**	EGF	EGF	**rs4444903**	4	3.69E-01	-0.21	1.35E-05	1.86E-04	-0.13	6.04E-02	3.49E-01
**Epidermal growth factor**	EGF	EGF	**rs4698756**	4	2.76E-01	-0.22	3.03E-05	4.02E-04	-0.11	1.53E-01	5.53E-01
**Epidermal growth factor**	EGF	EGF	**rs9992755**	4	3.75E-01	-0.20	1.27E-04	1.58E-03	-0.11	1.53E-01	5.53E-01
**Epidermal growth factor**	EGF	EGF	**rs2237051**	4	6.92E-01	-0.18	3.05E-04	3.67E-03	-0.15	3.33E-02	2.38E-01
**Epidermal growth factor receptor**	EGFR	EGFR	**rs13244925**	7	3.97E-01	-0.06	2.37E-02	1.45E-01	-0.03	2.75E-01	6.62E-01
**Epidermal growth factor receptor**	EGFR	EGFR	**rs10228436**	7	2.75E-01	-0.05	8.07E-02	3.09E-01	-0.09	2.83E-03	3.06E-02
**Epidermal growth factor receptor**	EGFR	EGFR	**rs2017000**	7	9.98E-02	-0.03	3.55E-01	6.75E-01	-0.09	3.48E-03	3.57E-02
**Epidermal growth factor receptor**	EGFR	EGFR	**rs9692301**	7	1.78E-01	0.00	8.98E-01	9.68E-01	0.06	4.78E-02	3.07E-01
**Erythropoietin**	EPO	EPO	**rs1734908**	7	9.14E-02	0.23	4.41E-02	2.11E-01	-0.07	5.58E-01	8.62E-01
**E-selectin**	E Selectin	SELE	**rs12027035**	1	2.78E-01	0.17	5.05E-02	2.31E-01	0.05	6.56E-01	8.93E-01
**Ferritin heavy chain**	FRTN	FTH1	**rs760306**	11	3.43E-01	-0.34	5.05E-02	2.31E-01	-0.06	7.21E-01	9.11E-01
**Glutathione S-transferase A1**	GST alpha	GSTA1	**rs4715332**	6	3.75E-01	0.53	9.25E-04	1.01E-02	0.27	1.43E-01	5.33E-01
**Granulocyte colony-stimulating factor**	G CSF	CSF3	**rs2302776**	17	7.26E-01	0.17	3.72E-02	1.97E-01	0.11	2.85E-01	6.76E-01
**Hepatitis A virus cellular receptor 1**	KIM 1	HAVCR1	**rs2279804**	5	6.32E-01	0.17	4.45E-02	2.11E-01	0.11	3.14E-01	6.89E-01
**Insulin-like growth factor-binding protein 2**	IGFBP 2	IGFBP2	**rs9341105**	2	3.01E-01	-0.17	3.75E-02	1.97E-01	-0.06	4.73E-01	7.91E-01
**Interleukin-1 receptor antagonist protein**	IL-1ra	IL1RN	**rs315919**	2	1.79E-01	-0.07	3.20E-01	6.33E-01	-0.23	1.69E-02	1.41E-01
**Interleukin-1 receptor antagonist protein**	IL-1ra	IL1RN	**rs3087266**	2	2.16E-01	-0.07	4.63E-01	7.50E-01	-0.27	4.85E-02	3.08E-01
**Interleukin-12 subunit beta**	IL-12p40	IL12B	**exm-rs3213094**	5	9.44E-01	-0.21	3.19E-02	1.77E-01	-0.27	4.22E-02	2.77E-01
**Interleukin-12 subunit beta**	IL-12p40	IL12B	**rs3212220**	5	9.44E-01	-0.21	3.19E-02	1.77E-01	-0.27	4.22E-02	2.77E-01
**Interleukin-18**	IL-18	IL18	**exm-rs1834481**	11	2.38E-01	0.16	1.53E-03	1.57E-02	0.07	2.24E-01	6.30E-01
**Interleukin-18**	IL-18	IL18	**rs5744256**	11	2.38E-01	0.16	1.53E-03	1.57E-02	0.07	2.24E-01	6.30E-01
**Interleukin-6 receptor**	IL-6r	IL6R	**exm-rs4129267**	1	6.24E-01	-0.44	4.64E-16	2.27E-14	-0.50	1.73E-15	3.67E-13
**Interleukin-6 receptor**	IL-6r	IL6R	**exm-rs4537545**	1	8.50E-01	-0.42	1.43E-15	6.08E-14	-0.45	1.13E-13	1.80E-11
**Interleukin-6 receptor**	IL-6r	IL6R	**rs4537545**	1	9.74E-01	-0.42	1.43E-15	6.08E-14	-0.44	5.47E-13	3.87E-11
**Interleukin-6 receptor**	IL-6r	IL6R	**exm-rs4845625**	1	9.54E-02	0.30	3.62E-09	6.58E-08	0.42	1.02E-11	5.88E-10
**Interleukin-6 receptor**	IL-6r	IL6R	**rs7514452**	1	5.06E-01	0.31	1.83E-07	2.98E-06	0.29	1.88E-04	2.45E-03
**Interleukin-6 receptor**	IL-6r	IL6R	**rs2229238**	1	5.13E-01	0.30	3.03E-07	4.82E-06	0.28	2.07E-04	2.63E-03
**Interleukin-6 receptor**	IL-6r	IL6R	**rs4240872**	1	6.98E-01	0.29	3.66E-07	5.68E-06	0.28	1.67E-04	2.21E-03
**Interleukin-7**	IL-7	IL7	**rs17504422**	8	1.32E-01	0.11	6.64E-01	8.59E-01	0.66	2.96E-02	2.24E-01
**Lymphotoxin-alpha**	TNF-beta	LTA	**exm-rs2009658**	6	6.72E-01	-0.30	4.59E-02	2.16E-01	-0.17	3.44E-01	7.14E-01
**Macrophage migration inhibitory factor**	MIF	MIF	**rs875643**	22	4.85E-01	-0.19	3.53E-02	1.89E-01	-0.12	2.55E-01	6.53E-01
**Matrix metalloproteinase-9**	MMP-9 total	MMP9	**rs3918240**	20	1.12E-01	0.02	7.94E-01	9.25E-01	0.15	1.46E-02	1.27E-01
**Metalloproteinase inhibitor 1**	TIMP 1	TIMP1	**rs4824621**	23	5.10E-01	-0.04	1.20E-01	3.87E-01	-0.06	3.46E-02	2.44E-01
**Myoglobin**	Myoglobin	MB	**rs4821423**	22	1.58E-01	0.04	4.70E-01	7.50E-01	0.22	4.20E-02	2.77E-01
**Osteopontin**	Osteopontin	SPP1	**rs6813526**	4	3.82E-01	0.03	7.01E-01	8.74E-01	0.19	2.64E-02	2.05E-01
**PAPf39**	PAP	ACPP	**rs9873881**	3	9.60E-01	-0.15	8.88E-03	6.76E-02	-0.14	8.01E-02	3.98E-01
**PAPf39**	PAP	ACPP	**rs3853148**	3	7.72E-01	-0.12	1.10E-02	7.85E-02	-0.15	1.93E-02	1.57E-01
**Plasminogen activator inhibitor 1**	PAI 1	SERPINE1	**rs1050955**	7	8.21E-02	0.04	4.03E-01	7.23E-01	0.17	4.69E-03	4.73E-02

**Probetacellulin**	BTC	BTC	**rs9307118**	4	1.57E-01	0.10	3.23E-02	1.77E-01	-0.04	6.60E-01	8.93E-01
**Proheparin-binding EGF-like growth factor**	HB EGF	HBEGF	**rs4150196**	5	3.76E-01	-0.11	4.17E-02	2.06E-01	-0.06	3.79E-01	7.44E-01
**Pro-interleukin-16**	IL-16	IL16	**rs11857713**	15	5.77E-01	0.49	1.13E-10	2.17E-09	0.44	2.18E-05	3.22E-04
**Pro-interleukin-16**	IL-16	IL16	**rs1995830**	15	4.64E-01	-0.17	1.92E-03	1.91E-02	-0.07	3.04E-01	6.89E-01
**Pro-interleukin-16**	IL-16	IL16	**rs11073001**	15	7.58E-01	0.14	8.99E-03	6.76E-02	0.18	1.18E-02	1.09E-01
**Pro-interleukin-16**	IL-16	IL16	**rs117196289**	15	9.29E-02	0.31	3.36E-02	1.83E-01	-0.18	4.57E-01	7.91E-01
**Pro-interleukin-16**	IL-16	IL16	**rs4589514**	15	4.79E-01	-0.13	3.93E-02	2.03E-01	-0.19	3.19E-02	2.33E-01
**Pro-interleukin-16**	IL-16	IL16	**rs4778641**	15	1.06E-01	0.02	6.88E-01	8.65E-01	0.13	2.49E-02	2.00E-01
**Serotransferrin**	Transferrin	TF	**exm-rs3811647**	3	3.25E-01	-0.10	1.79E-03	1.81E-02	-0.05	1.41E-01	5.33E-01
**Serotransferrin**	Transferrin	TF	**rs6762719**	3	4.24E-01	-0.09	4.51E-03	3.82E-02	-0.05	1.41E-01	5.33E-01
**Soluble KIT ligand**	SCF	KITLG	**rs1000788**	12	6.49E-02	-0.13	3.96E-02	2.03E-01	0.06	4.01E-01	7.57E-01
**Sortilin**	Sortilin	SORT1	**rs10858092**	1	4.44E-01	0.13	1.40E-02	9.30E-02	0.07	1.66E-01	5.65E-01
**Sortilin**	Sortilin	SORT1	**rs11102972**	1	5.92E-01	0.12	2.70E-02	1.63E-01	0.08	2.34E-01	6.42E-01
**Stromelysin-1**	MMP-3	MMP3	**rs679620**	11	7.05E-01	0.34	1.79E-08	3.09E-07	0.30	3.48E-05	5.03E-04
**Stromelysin-1**	MMP-3	MMP3	**rs566125**	11	3.02E-01	-0.32	2.63E-04	3.21E-03	-0.20	3.06E-02	2.29E-01
**Stromelysin-1**	MMP-3	MMP3	**rs522616**	11	6.90E-01	-0.23	1.49E-03	1.57E-02	-0.26	7.26E-03	7.11E-02
**Tenascin**	TN C	TNC	**rs2071520**	9	1.14E-01	-0.16	4.39E-03	3.77E-02	-0.01	9.16E-01	9.88E-01
**Tenascin**	TN C	TNC	**rs953288**	9	2.37E-01	-0.14	5.99E-03	4.82E-02	-0.04	5.83E-01	8.67E-01
**Tenascin**	TN C	TNC	**rs10122770**	9	2.66E-01	0.33	6.64E-03	5.28E-02	0.08	6.29E-01	8.89E-01
**Tenascin**	TN C	TNC	**rs7847271**	9	1.61E-01	0.21	2.85E-02	1.69E-01	-0.03	7.79E-01	9.32E-01
**Tenascin**	TN C	TNC	**rs1330368**	9	7.10E-01	-0.11	3.94E-02	2.03E-01	-0.07	2.72E-01	6.62E-01
**Tenascin**	TN C	TNC	**rs1547691**	9	9.36E-01	-0.12	4.39E-02	2.11E-01	-0.10	1.88E-01	5.91E-01
**Thrombospondin-1**	Thrombospondin 1	THBS1	**rs2169830**	15	1.32E-01	-0.16	5.19E-02	2.36E-01	0.03	7.51E-01	9.20E-01
**Thyroxine-binding globulin**	TBG	SERPINA7	**rs1804495**	23	2.92E-01	0.19	2.33E-03	2.28E-02	0.11	8.56E-02	4.12E-01
**Tumor necrosis factor receptor superfamily member 6**	FAS	FAS	**rs1977389**	10	6.73E-01	-0.10	2.31E-02	1.43E-01	0.00	9.89E-01	9.94E-01
**Tumor necrosis factor, soluble form**	TNF-alpha	TNF	**exm-rs1800629**	6	5.56E-02	0.28	1.39E-02	9.29E-02	-0.07	6.47E-01	8.91E-01
**Uromodulin, secreted form**	THP	UMOD	**rs11647727**	16	3.52E-01	0.53	7.81E-15	3.10E-13	0.45	2.26E-07	4.95E-06
**Uromodulin, secreted form**	THP	UMOD	**exm-rs12917707**	16	5.91E-01	0.61	7.26E-14	2.72E-12	0.68	2.96E-13	2.35E-11
**Uromodulin, secreted form**	THP	UMOD	**rs4293393**	16	4.95E-01	0.60	1.14E-13	4.03E-12	0.68	2.96E-13	2.35E-11
**Uromodulin, secreted form**	THP	UMOD	**exm-rs4293393**	16	4.61E-01	0.59	1.52E-13	5.08E-12	0.68	2.96E-13	2.35E-11
**Uromodulin, secreted form**	THP	UMOD	**exm-rs13333226**	16	3.83E-01	0.58	6.40E-13	2.03E-11	0.68	2.96E-13	2.35E-11
**Uromodulin, secreted form**	THP	UMOD	**rs9646256**	16	5.31E-01	0.37	7.15E-09	1.26E-07	0.30	1.26E-05	2.35E-04
**Uromodulin, secreted form**	THP	UMOD	**rs9652589**	16	3.98E-01	-0.14	3.22E-02	1.77E-01	-0.22	1.93E-03	2.20E-02
**Uromodulin, secreted form**	THP	UMOD	**exm-rs12444268**	16	6.76E-01	0.12	1.20E-01	3.87E-01	0.17	3.23E-02	2.33E-01
**Vasostatin-1**	CgA	CHGA	**rs9658634**	14	1.75E-01	-0.21	2.82E-01	5.91E-01	-0.61	1.45E-02	1.27E-01

Key: Interaction P-value, SNP x Diagnosis interaction significance.

Supplementary figure 1. Scatterplots showing significant SNP and protein expression associations where SNP x Diagnosis interaction was significant

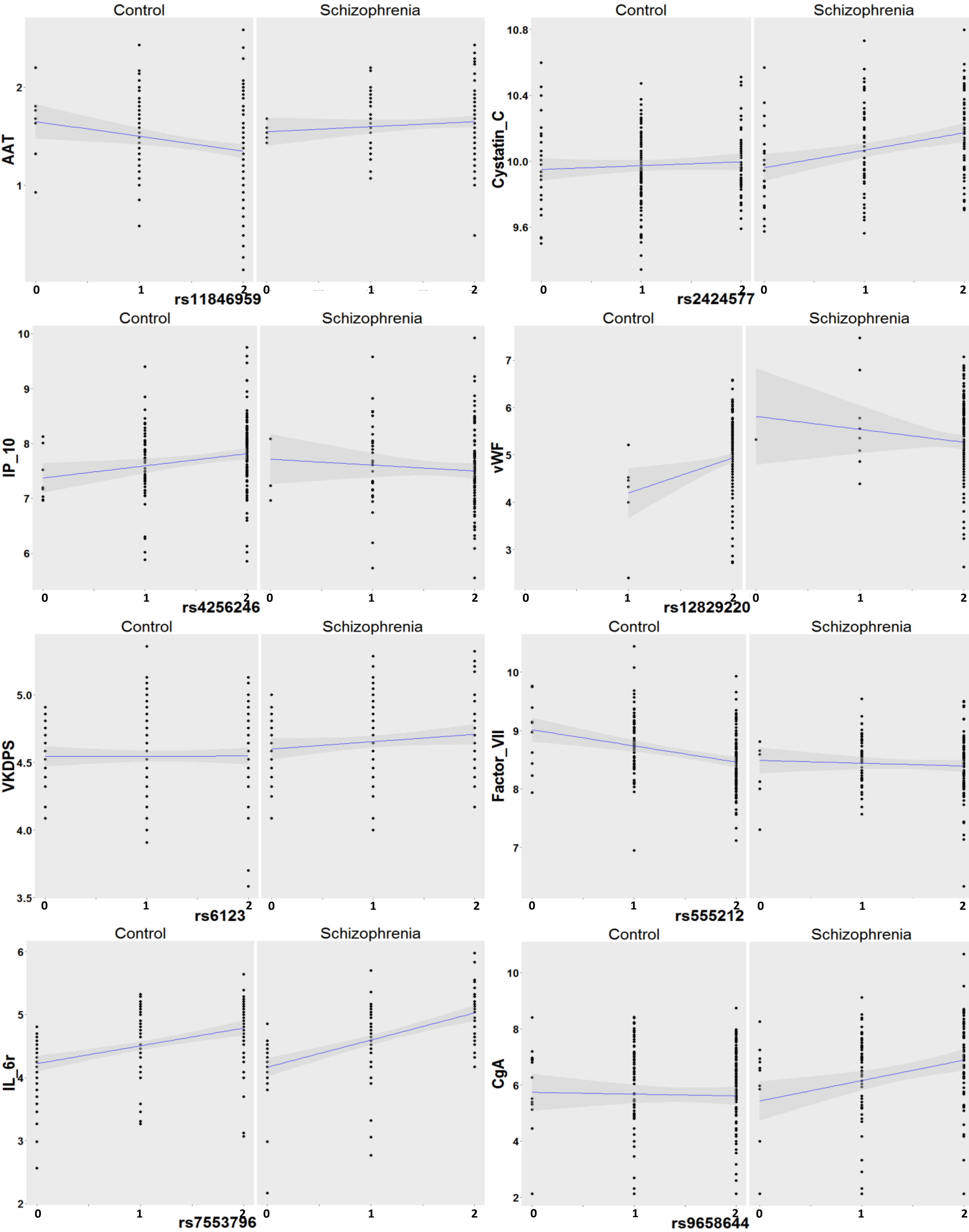

Supplementary Figure 2. Graphical illustration of the effect of genetic variation on protein biomarker expression in blood and differential regulatory pathways involved

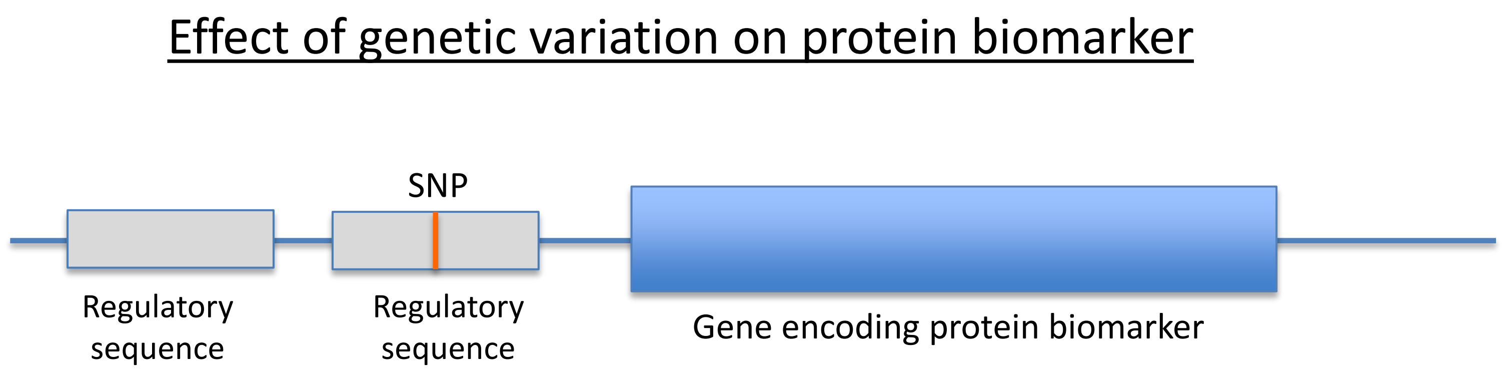

Effect of genetic variation on protein biomarker independent of disease status:

Regulatory pathways are identical in disease and health

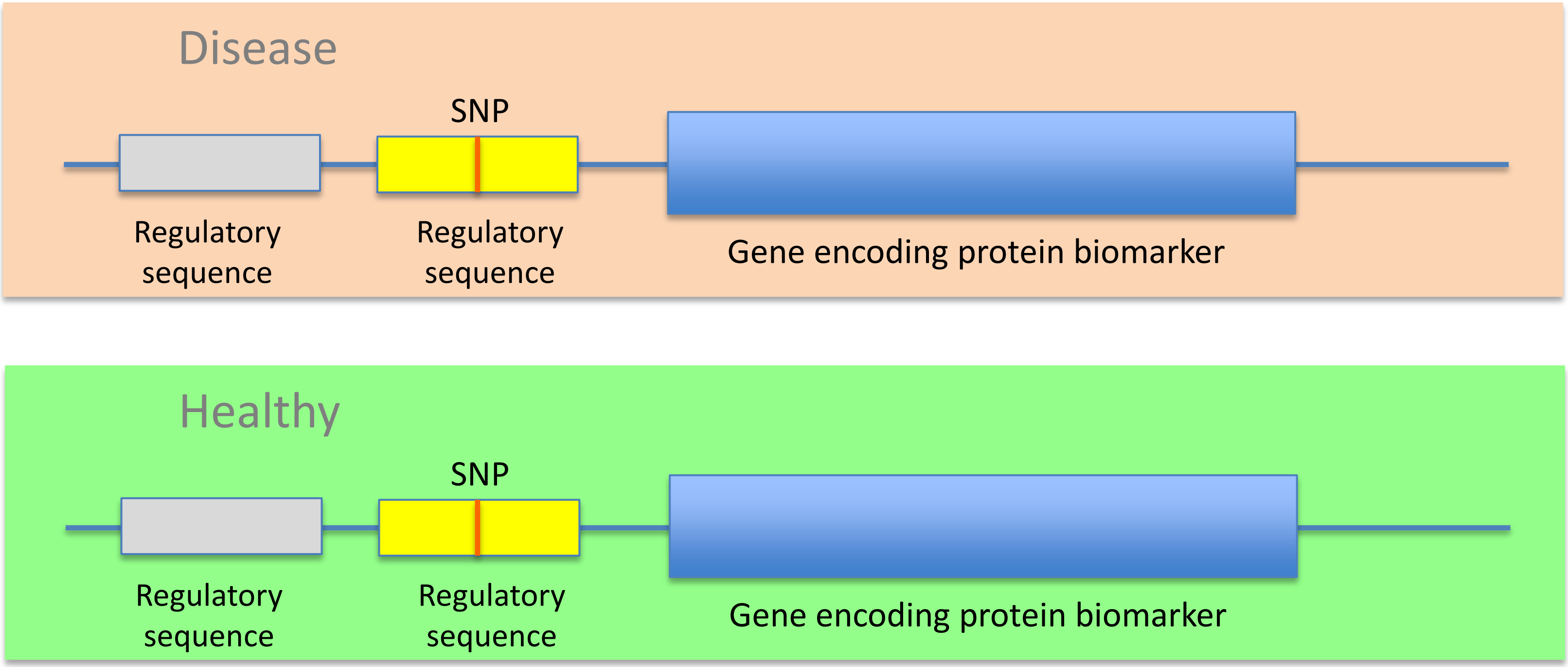

Effect of genetic variation on protein biomarker dependent of disease status: Regulatory pathways are different in disease and health

A) Effect of genetic variation present in patients and not in healthy individuals -> the SNP containing regulatory sequence is involved in a disease-specific process

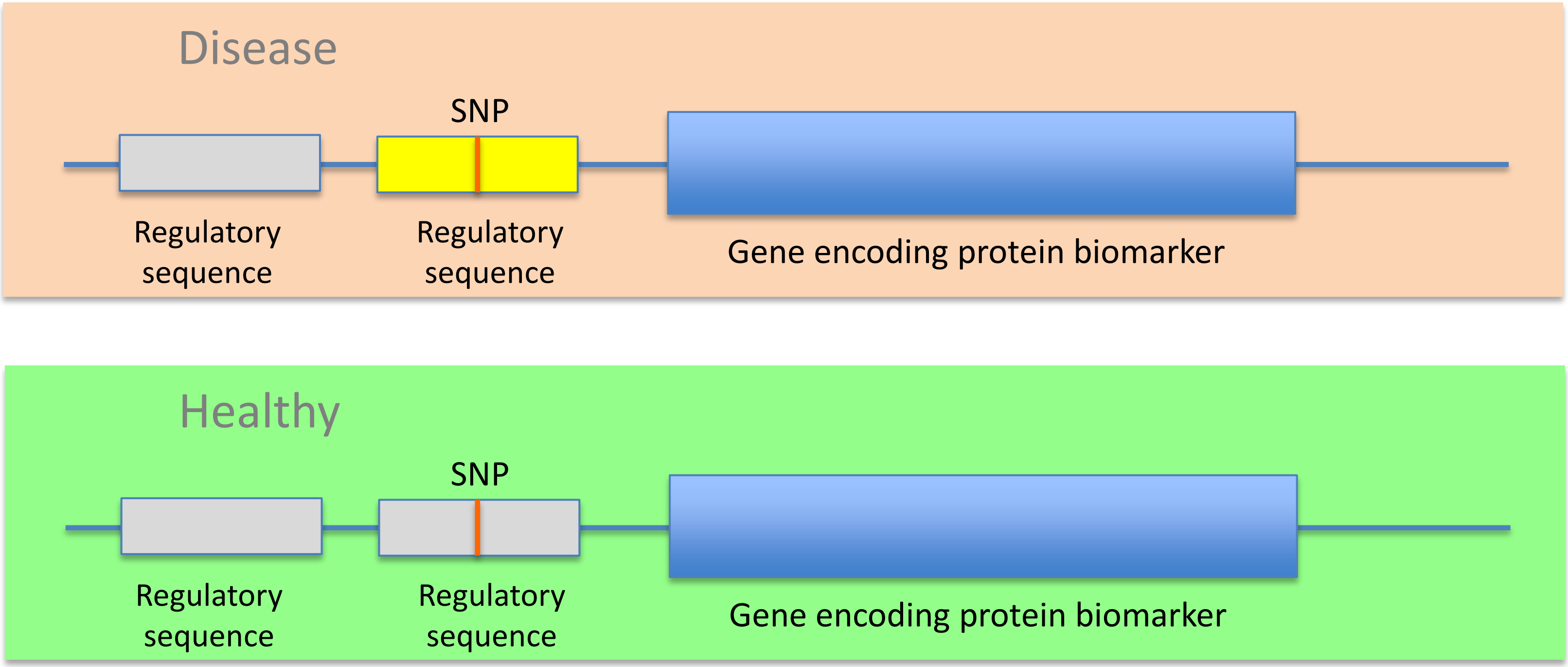

Effect of genetic variation on protein biomarker dependent of disease status: Regulatory pathways are different in disease and health

B) Effect of genetic variation present in healthy individuals and not in patients -> the SNP containing regulatory sequence is involved in a health-specific process

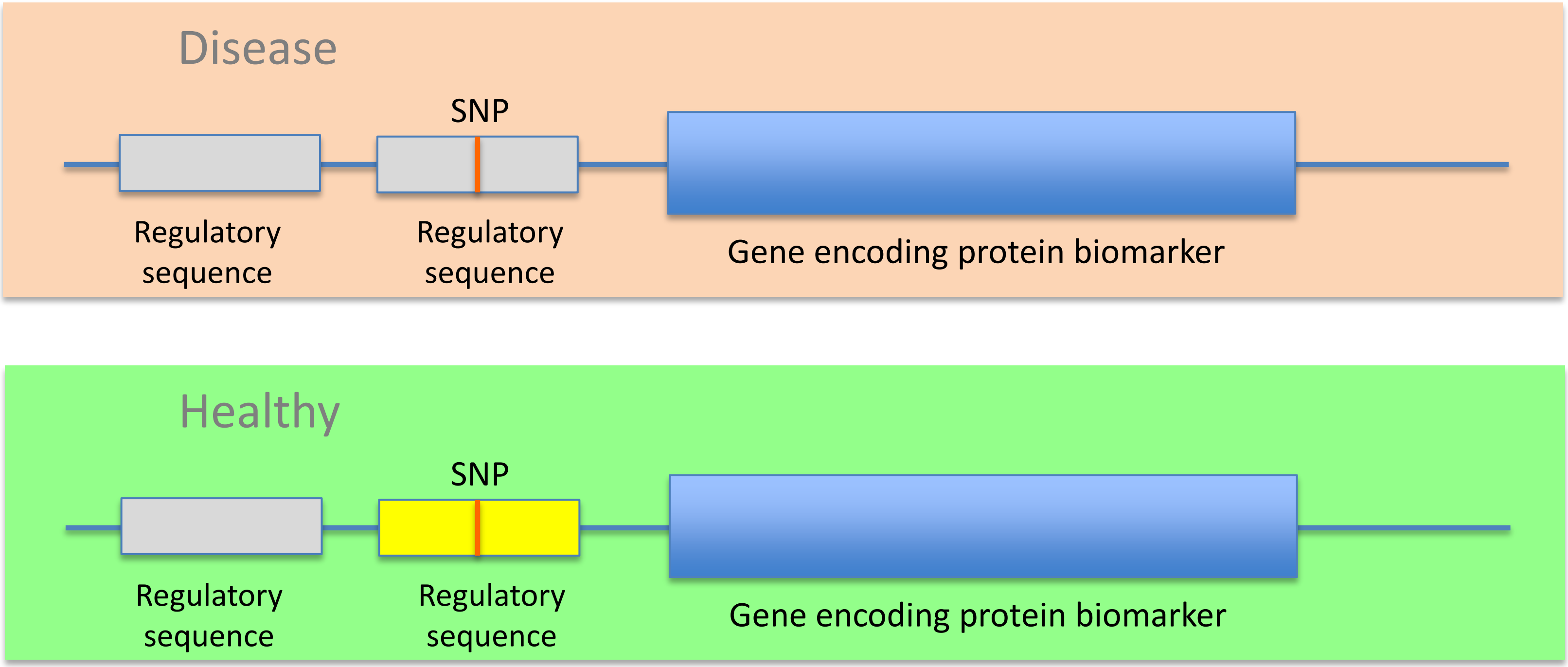

Supplement: Supplementary file 1 — Supplementary Material [file 41598_2017_12986_MOESM1_ESM.pdf]
